# Air–Liquid Interface *In Vitro* Models for Respiratory Toxicology Research: Consensus Workshop and Recommendations

**DOI:** 10.1089/aivt.2017.0034

**Published:** 2018-06-01

**Authors:** Ghislaine Lacroix, Wolfgang Koch, Detlef Ritter, Arno C. Gutleb, Søren Thor Larsen, Thomas Loret, Filippo Zanetti, Samuel Constant, Savvina Chortarea, Barbara Rothen-Rutishauser, Pieter S. Hiemstra, Emeric Frejafon, Philippe Hubert, Laura Gribaldo, Peter Kearns, Jean-Marc Aublant, Silvia Diabaté, Carsten Weiss, Antoinette de Groot, Ingeborg Kooter

**Affiliations:** ^1^ Chronic Risks Division, Institut National de l'Environnement Industriel et des RISques, Verneuil-en-Halatte, France.; ^2^ In Vitro und Mechanistische Toxikologie, Fraunhofer ITEM, Hannover, Germany.; ^3^ Environmental Research and Innovation (ERIN) Department, Luxembourg Institute of Science and Technology, Esch-sur-Alzette, Luxembourg.; ^4^ Inhalation Toxicology Group, National Research Centre for the Working Environment, Copenhagen, Denmark.; ^5^ Systems Toxicology Department, Philip Morris International R&D, Neuchâtel, Switzerland.; ^6^ Epithelix Sarl, Plan-les-Outes, Switzerland.; ^7^ BioNanomaterials, Adolphe Merkle Institute, University of Fribourg, Fribourg, Switzerland.; ^8^ Laboratory for Materials-Biology Interactions, EMPA, Swiss Federal Laboratories for Materials, Science and Technology, St Gallen, Switzerland.; ^9^ Department of Pulmonology, Leiden University Medical Center, Leiden, The Netherlands.; ^10^ Directorate F-Health, Consumers and Reference Materials Chemicals Safety and Alternative Methods Unit (F.3), EURL ECVAM, JRC, Ispra, Italy.; ^11^ Environment, Health and Safety Division, OECD, Paris, France.; ^12^ European Affairs and Standardization, Laboratoire National de Métrologie et d'Essais, Paris, France.; ^13^ Institute of Toxicology and Genetics, Karlsruhe Institute of Technology, Karlsruhe, Germany.; ^14^ Toxicological and Environmental Risk Assessment (TERA) Department, Solvay, Brussels, Belgium.; ^15^ Department of Circular Environment and Environment (CEE), TNO, Utrecht, The Netherlands.

**Keywords:** air–liquid interface, *in vitro*, inhalation, lung cell models, toxicology, validation

## Abstract

*In vitro* air–liquid interface (ALI) cell culture models can potentially be used to assess inhalation toxicology endpoints and are usually considered, in terms of relevancy, between classic (i.e., submerged) *in vitro* models and animal-based models. In some situations that need to be clearly defined, ALI methods may represent a complement or an alternative option to *in vivo* experimentations or classic *in vitro* methods. However, it is clear that many different approaches exist and that only very limited validation studies have been carried out to date. This means comparison of data from different methods is difficult and available methods are currently not suitable for use in regulatory assessments. This is despite inhalation toxicology being a priority area for many governmental organizations. In this setting, a 1-day workshop on ALI *in vitro* models for respiratory toxicology research was organized in Paris in March 2016 to assess the situation and to discuss what might be possible in terms of validation studies. The workshop was attended by major parties in Europe and brought together more than 60 representatives from various academic, commercial, and regulatory organizations. Following plenary, oral, and poster presentations, an expert panel was convened to lead a discussion on possible approaches to validation studies for ALI inhalation models. A series of recommendations were made and the outcomes of the workshop are reported.

## Introduction

Air–liquid interface (ALI) *in vitro* lung cell models can be used to investigate physiological and pathophysiological responses of the respiratory tract, molecular events, and modes of action and interaction of different cell types. Potentially, they mimic more closely the *in vivo* situation of cells in the respiratory tract, namely, being apically exposed to air. Such ALI models focus on particular anatomical regions of the lung or particular molecular pathways and aim to model processes that are relevant *in vivo.*^1,2^

Interest in such models has grown since they are under development to provide information on the effects of xenobiotics in lungs and potentially downstream systemic consequences that may relate to health and disease endpoints. Such observed effects may be relevant for the *in vivo* situation and the models may therefore also have the potential to reduce the numbers of animals used in experimental toxicology and risk/hazard assessments required in many jurisdictions especially if international validation (via The European Union Reference Laboratory for Alternatives to Animal Testing, i.e., EURL ECVAM) could be achieved. Animals have routinely been used for such assessments simply because no *in vitro* methods have ever been validated to sufficient levels. In that respect, animal usage in this domain is still widely seen as the only option available. However, anatomical differences between rodent and human lungs are significant, which has strong implications for the deposition rates and localizations of all particulates making extrapolation from animals to humans difficult. There is a strong societal and legal support to reduce, refine, and replace the use of animals (3Rs) and this in turn is driving the development of so-called alternative methods—the most promising being ALI approaches in the domain of inhalation toxicology.

Verification or validation of alternative methods is therefore seen as key to progress in the field of inhalation toxicology. To this end, data from different *in vitro* experimental approaches need to be compared and contrasted with *in vivo* studies.^3^ Unfortunately, at the moment, no alternative method that specifically uses respiratory tract cells cultured under ALI conditions and exposed via air is validated by EURL ECVAM and there is a clear lack of harmonization/standardization in the way such methods are currently used. This is a major problem for hazard and risk assessment as the reliability and reproducibility of results and hence the conclusions that can be drawn from such experiments are at present rather limited.

Against this background, a workshop on ALI *in vitro* models for respiratory toxicology research was organized in Paris on March 21, 2016, to bring together the major parties in Europe with a stake in the use of ALI *in vitro* lung cell models. The workshop was organized under the auspices of NanoReg2, a European Union (EU)-funded Horizon 2020 research and innovation program in conjunction with INERIS, France, and TNO, The Netherlands.

In total, around 60 scientists from academia, industry, and regulatory bodies attended the 1-day workshop. The main aims were to discuss the current state-of-the-art exchange experience in terms of practical aspects and to discuss how to move forward with the air–liquid approaches with regard to future implementation in a regulatory context. The workshop consisted of a series of plenary lectures, short oral presentations, poster presentations, and finally, a discussion led by a panel of experts. This article documents the outcomes of the workshop and provides a series of recommendations for the future development of the methods either in the main text or in the Supplementary Data (Supplementary Data are available online at www.liebertpub.com/aivt).

After presenting background information, the article deals with the current knowledge and practices of ALI approaches, in terms of atmosphere generation, exposure principles, and cell models available. Then, the outcomes of the expert panel discussion are presented, highlighting the validation process and the challenges to overcome the validation of ALI techniques. Finally, several recommendations are presented to implement the validation process.

## Background

Respiratory health and disease related to ambient air pollution are a major concern worldwide.^4^ Apart from air pollution, numerous chemicals and particles need to be assessed in the context of the EU REACH (registration, evaluation, authorisation, and restriction of chemicals) regulation for pulmonary toxicity. Health effects due to inhalation of substances are therefore of considerable interest to many and form the basis for the use and development of *in vitro* models of the respiratory tract. As well as studying the efficacy of new pharmaceutically active compounds,^5^ a major use of the models is to examine the potentially toxic effects of different types of compounds ranging from chemicals to (nano)particles, such as Ag,^6^ SiO_2_,^7^ CeO_2_, and TiO_2_,^8^ multiwall carbon nanotubes,^9^ or complex combustion-derived aerosols.^10–12^

Many different models have been used ranging from *in vivo* animal experiments to *in vitro* models using lung cells.^13^ In the latter case, many different types of exposure scenarios have been used and indeed the cells used can be animal or human derived. Despite differences between the various models, the core ambition is to model what likely happens *in vivo* as closely as possible with *in vitro* techniques.

ALI *in vitro* inhalation models are increasingly being used in research and this comes with some advantages, not least that their use has the potential to reduce the numbers of animals used in research because they are more realistic as they mimic more closely the pulmonary region than classic (i.e., submerged) *in vitro* methods. Their use may also provide better information on *in vivo* processes in humans and allow investigations into mechanisms that occur in humans (some of which might differ or do not exist in animal models due to variations in physiology).^14^

Their increasing use in EU-funded projects (e.g., FUTURENANONEEDS, npSCOPE, SPOTVIEW, NANOREG, NANOREG2, PneumoNP) highlights the levels of interest in ALI models and potential realms of application. While their increased use is starting to provide novel information on endpoints and potential mechanisms, stakeholders at the EU level have raised questions about levels of validation and standardization of the methods and concerns about how much information coming from the projects in particular can actually be compared. On the basis of this, the suggestion was made by one particular stakeholder, to pull together relevant experts to discuss whether and how such validation and standardization studies could be performed.

The workshop in Paris in March 2016 was the result and had the specific aim of starting a process of alignment among stakeholders toward the validation of *in vitro* ALI inhalation lung cell models combined with the air–liquid exposure system to aerosolize a substance/chemical of interest.

## ALI Exposure: Recent Advances and Challenges

### Generation of exposure atmospheres for biological applications

#### General considerations

The purpose of delivery of inhaled substances to cells via an ALI is to mimic the physiological processes of mass transfer to and within the epithelial layers of the lung.

For particles, the mass transfer to the surfaces is determined by directional volume forces such as gravity, thermophoretic and electrical forces, as well as diffusional motion. The transfer rates are strongly dependent on the aerodynamic size of the particles.

The transport of vapors to the surfaces is primarily controlled by the interaction of the substance with the liquid lining layer/cell surface. This usually occurs via dissolution and reactivity processes.

To be comparable with real-life exposure, the composition of the exposure atmosphere in the test setup should be similar to what it is in real life regarding particle size distribution and chemical composition. The determination of administered dose is required for the interpretation and classification of measured biological effects. For particles, the dose can be measured in a reference membrane insert either equipped with a quartz crystal balance or by chemical and fluorescence analysis of the material recovered from the insert.^15^ Modeling can also contribute to dose determination (see e.g., Refs.^16,17^).

Methods for the generation of exposure atmospheres to be tested with cell-based *in vitro* methods can be set up for gases, vapors, complex atmospheres such as from diesel engines, wood stoves, or others, or from pure test materials.

#### Methods of aerosol generation from pure test materials

There are three different ways to generate aerosols: formation of aerosols by dispersion of liquids, generation of dry solid particles by dispersion of powders or ablation from solid bulk materials, or particle formation by gas to particle formation (i.e., nucleation and subsequent growth by condensation and coagulation).^18–21^

##### Liquids

Liquid drops can be generated using the inherent instabilities of filaments and jets, which are formed by application of hydrodynamic forces applied to the liquid in various nozzle configurations.^22^ Frequently, air-assisted nebulizers are used to generate aerosols for medical application. The liquid mass flux is either established by the Venturi principle or it is fed into the dispersion unit by a peristaltic pump allowing for independent control of the liquid mass flow. The liquid is disintegrated by interaction with high-speed airflow in the zone where the liquid flow and the air flow are mixed. Liquid dispersion can also be facilitated without airflow at all, either by using ultrasonic energy applied to a liquid surface, by pumping the liquid through an oscillating perforated mesh, or by using electrical forces. The underlying physical principles of droplet formation are the instability of capillary surface waves formed when the liquid is agitated by ultrasonic waves and the natural Rayleigh instabilities of the jets formed in the perforated mesh system. When a fluid jet is highly charged, Coulombic repulsion leads to droplet formation. This method is called electrospraying.^23,24^

Depending on the process parameters, mass median aerodynamic droplet diameters in the range between 2 and 10 μm can be achieved using mechanical generation methods,^25^ whereas electrospray generation results in submicron droplets (<1 μm). The liquid generation method can be applied to pure liquids as well as to solutions of nonvolatile compounds or suspensions of nanoparticles. This offers the possibility to further reduce the aerosol size by evaporation of the solvent and will result in droplet shrinkage until a dry aerosol state is reached where it is composed of the nonvolatile compound only. This method is particularly interesting when nanoaerosols with a low degree of agglomeration have to be produced, which can be achieved by electrospraying dispersions of low concentrations of solid material (as shown by Fu et al.^23^). Here the initial droplets are already in the 100 nm range. After evaporation of the water, the dry aerosol particles can be as small as several nanometers.

##### Powders and bulk material

Generation of aerosols from dry powders is somewhat similar to nebulization of liquids in that it requires a feeding technology to establish the powder mass flow and the supply of external energy for its deagglomeration. Powder feeding can be metered by various mechanisms that include spiral screws, groves, or holes in a rotating disk, or just gravitational forces. Dispersion is facilitated by stationary or transient high air speeds,^26–29^ scraping mechanisms, or mutual collisions between powder agglomerates such as jet mills, fluidized beds,^30,31^ externally excited fluidized beds (vortex shaker^32^), or collisions of agglomerates with surfaces (fluidized bed with dispersion balls). Methods based on acoustic excitations of a powder layer are used particularly for the generation of airborne fibrous materials such as carbon nanotubes.^33^

Aerosols can also be generated directly from solid bulk materials by electrical spark ablation or laser ablation. In the spark generator, molecules or clusters are detached from the surfaces of solid bodies by electrical sparks established between two electrodes made of the solid compound under consideration. Therefore, this method is limited to conductive materials. Laser ablation requires the absorption of light by the compounds. The spark generator is widely applied in toxicity studies particularly in view of elucidating the mechanistic aspects of any biological effects.^34,35^ Laser ablation is a well-suited method to produce nanoparticles directly in the aqueous environment. This suspension can then be aerosolized using the methods described in the previous section.^36^

##### Gas to particle conversion

A completely different route for (nano-) aerosol generation is gas to particle conversion. Combustion-generated aerosols are formed in this way. Typically, there are gaseous precursors at elevated temperatures, which form condensable species either just by cooling or by chemical reaction. Particles then grow by heterogeneous condensation and/or coagulation. By properly quenching the processes, particle distributions can be limited to the ultrafine size regime. Pure condensational growth leads to nearly monodisperse particles, whereas coagulation results in wider particle size distributions.

Gold particles formed by gas to particle conversion, which then interact with various biological fluids, have previously been described.^37^ A method to generate zinc oxide particles in a flame reactor has also been described, and in this case it was demonstrated that using the submerged and ALI routes of cell exposure did result in different (toxicological) endpoints being reached.^38^ A method has also been developed to examine inhalational exposure to bitumen fumes based on the evaporation and recondensation of fume condensates sampled from bitumen storage tanks.^39^ This approach effectively allows one to exactly mimic real fume exposure at specific workplaces and to apply the resulting aerosol in both *in vivo* and *in vitro* experiments.

ALI exposure units have to be properly interfaced with the generation method. This can be done by placing a mixing/equilibration volume between generation and exposure system. The volume flows of the aerosol generation system can be decoupled from the airflow used for cell exposure. A feedback control system can be helpful to keep the concentration at the desired level.

### Exposure of cell-based *in vitro* systems to study biological effects of airborne compounds

#### General principles/background on *in vitro* airborne exposure

The exposure itself has in principle one simple but major task: to establish an effective contact between the biological test system used, such as cells,^40^ tissue, or *ex vivo* material (e.g., precision-cut lung slices^41^), and the exposure atmosphere. This includes an intensive and defined contact with the gas phase as well as with the particle phase of airborne material and to establish dose metrics as a fundamental requirement of any successful *in vitro* testing.

Moreover, any artificial exposure effects on the exposed biological material have to be avoided. Since the original environment of any biological test system is the liquid phase whereas the original environment of the exposure atmosphere is by definition the gas phase, there are mainly three possible ways to go for a combination:

(1) by transferring the test material from the gas phase to the liquid phase,
(2) by transferring the biological test system to the gas phase for a time intermittently to (1), above, or,
(3) by transferring the biological test system to the gas phase continuously.

In reality, these three strategies have been applied during previous decades to establish cell-based *in vitro* methods for testing airborne material. Already more than 40 years ago, cell suspensions from alveolar macrophages were used to study the biological effect of chemical gases such as nitrogen dioxide by bubbling the gas through the culture medium.^42^

This strategy is still used for setting up new methods such as the hanging-drop exposure model.^43^ Here human lung cells (A549 cell line) are used in suspension culture to then be exposed to gaseous chemicals inside a small drop under static exposure conditions in a closed vessel. Strategies corresponding to (2) (above) are represented by intermittent exposures of cells or *ex vivo* material to the atmosphere using half-filled roller bottles^44,45^ or tilted, rotating multiwell plates.^46^

With respect to the key feature of biological relevance, it is possible that the cellular organization of the biological cell or tissue material used in such approaches may be relatively far away from the *in vivo* situation in the human lung.

Also, with respect to exposure relevance, it has to be taken into account that the presence of culture medium as the compound of primary contact with the test material will result in a relatively low efficiency of contact between the test atmosphere and the cells.^47^ Moreover, it will lead to physicochemical modifications of the airborne test material. In the simplest case this will lead to a change in particle sizes. However, depending on the test material, it may also lead to chemical changes in the test atmosphere in case of chemically reactive components in a complex mixture.^48^

Also, exact dose can be hard to accurately measure due to unknown physicochemical properties and kinetics of the test material in specific culture media. As a result, these strategies do not represent the primary route of exposure of the inner biological surfaces of the human respiratory system to inhaled atmospheres.

#### ALI exposure

Contrasting to this, cultures lifted to the ALI^42^ seem to offer the basis for a perfect translation of the biological *in vivo* organization of the respiratory system to *in vitro*. By culturing tissue, cells, or *ex vivo* material on microporous membranes while feeding and humidifying them only from the basal side, a biological barrier model is set up separating the inhaled air/exposure atmosphere compartment from a blood/liquid/culture medium compartment in a construction that is highly relevant to the *in vivo* setting.

With respect to exposure relevance, ALI models offer unique properties.

Due to the exposure situation of the models that expose the biological system continuously to the atmosphere, the exposure agent does not interact with cell culture medium, which, especially for particles, can result in changes of the properties (dissolution, agglomeration).

As a result, it is possible to conduct exposure approaches from real-life atmospheres by taking the exposure setup, including the biological test system to the source of the exposure atmosphere.^49^ This is also the experimental basis to consider relevant factors such as physicochemical properties of the test atmosphere, different experimental methods for generation and characterization of test aerosols and to establish an unhindered, defined contact between the biological test system and the test material in the airborne state. Hence, also from the point of exposure relevance, ALI models exhibit a large range of possibilities.

The ALI exposure situation described above could be seen as an “ALI microclimate” condition. Two main mass transports control this state: the mass flow of liquid culture media through the membrane toward the cells and the fluid flow necessary to transport the airborne test material to the surface of the biological test system.

A range of setup specific factors can then have a large impact on this scenario. Cell-specific characteristics (e.g., the formation of cell/cell contacts that provide epithelial barrier activity), pore sizes, and pore densities of the membrane, hydrophobicity of the membrane surface, flow rate of the gas flux, humidification of the test atmosphere, pressures of culture media and test atmosphere, characteristics of the culture media (e.g., osmolarity, viscosity) and more are all examples.

By appropriately setting up these parameters, it is possible to generate an “in control” state, where high exposure efficiencies and a good preservation of cellular viability can be expected at the same time. However, any physical coverage of cells has to be taken into account as an important factor of influence on the system. Types of cell coverage may include the intended presence of mucus, epithelial lining fluid, or a surfactant, and these are usually included to increase the relevance of the biological model. Cell coverage may also occur in an unintended manner from, for example, an increased flux of culture media through the membrane as a result of the chosen membrane type and pore size.

In any case, cell coverage by fluids will have a large impact on the sensitivity and relevance of the model, since a physicochemical cross talk between the coverage and the airborne test material will now take place. Hence, diffusion kinetics and reaction kinetics will be limiting for the contact of cells and the gas/aerosol phase. As an example, it is well known^48^ that only very small coverages of biological materials by lining fluids prevent contact of ozone with cells and that this is due to the extremely fast reaction kinetics of ozone. This can also be shown experimentally through variation of cell coverage by culture media during ALI exposure—there is a clear change in dose/response of cells toward ozone.^47^ Hence, a definition of the specific ALI exposure state is critically needed for specific exposure setups, especially when setups using different biological test systems are to be compared with respect to the outcome of a testing scenario. It is especially important to consider the epithelial barrier activity of the culture used, to prevent medium leakage to the apical surface of the cultured cell layer, which may affect the outcome of the exposure.

**FIG. 1.**
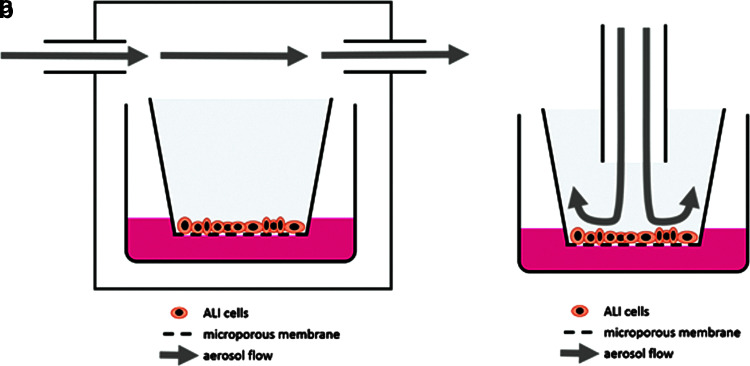
Main ALI exposure setups. **(a)** “Incubator/box-type” setup featuring a horizontal flow, a “smooth” cell exposure, and often using standard commercial culture plates. It results in a conjoined exposure of cultures with less defined fluid dynamics. **(b)** “Stagnation point flow” setup featuring individual exposure of single cultures, offering optimized fluid dynamics and an effective cell to gas contact although it usually calls for custom-made constructions and more sophisticated setups. ALI, air–liquid interface.

The flow alignment of ALI exposure setups is usually one of two types (Fig. 1). The incubator/box-type setup^50–52^ includes the positioning of cultures inside a box, which may also be a cell culture incubator. The box is then filled or rinsed with the test atmosphere leading to a horizontal exposure flow above the inserts of the cultures. A more sophisticated setup is represented by the stagnation flow setup^53,54^ where the exposure flow is directed toward the ALI surface. Using this flow alignment, an individual exposure of single cultures can be achieved. Moreover, an optimization of fluid dynamics is possible leading to a better-defined dosimetry and a higher exposure efficiency. Exposure setups based on these basic ALI flow alignments are commercially available and can be found in the literature or as in-house solutions of a number of working groups. Hence, for example, TSE Systems (Bad Homburg, Germany) is offering an exposure system based on a box, while others^55,56^ have published respective setups based on box designs. The same is true for the basic stagnation flow setup, where in-house versions^57^ and commercial solutions are available (e.g., Vitrocell, Waldkirch, Germany; CULTEX, Hannover, Germany, among others).

Using these basic exposure alignments, exposures of cells to gases have been successfully conducted, and as a result, a first small prevalidation study has been performed in Germany.^58^ There was good inter- and intralaboratory reproducibility, and a relevance of the model using A549 cells could be shown for acute toxicity screening of chemical gases in terms of acting as a first predictive model.

#### Particle deposition

The exposure scenario becomes more complicated when particles are involved during exposure to aerosols. Particle deposition in the lung *in vivo* is a complex process and strongly affected by physicochemical properties of the test aerosol (e.g., particle characteristics such as size, shape, density, and solvability can all play a role). This leads to particle deposition rates that differ according to properties, and that also vary according to the lung region being studied. Such behavior can be estimated with mathematical models such as the ICRP (International Commission on Radiological Protection)^59^ or the MPPD (Multi-Path Particle Dosimetry) models.^60^

As a consequence of this, certain test aerosols, especially when composed of a mixture of different compounds, do not have an equal relevance for all lung regions. Hence, the translation of the *in vivo* scenario to an appropriate *in vitro* modeling scenario has not been fully addressed in many cases. Strategies to solve such issues can include aiming for a maximum deposition efficiency within the *in vitro* model independent of the particulate size under investigation. Alternatively, a specific deposition efficiency can be defined according to a selected localization in the lung or an *in vivo* curve-like deposition efficiency or others. Especially with regard to any standardization or harmonization projects, these basic strategies are of fundamental importance to achieve comparable exposure and dosimetry characteristics using different experimental approaches.

For individual experiments involving the *in vitro* exposure of cells to aerosols, the most important factor governing outcomes is the deposition efficiency of the aerosols onto the surface of cells. Its impact on outcomes can be limited, however, using basic flow alignments that alter sedimentation and diffusion mechanisms. Such approaches are available in many of the systems mentioned above. For example, it has been shown that the deposition efficiency of these systems is in the range of 2% for particle sizes smaller than 1000 nm.^54,61^ Hence, 98% of the particles conducted over the cellular surface during exposure are just lost. On that basis, it has been calculated^16^ what exposure time would be needed in an experimental approach, where the *in vivo* lowest observed adverse effect level determined in submerged experiments from silicon dioxide of 15.6 μg/cm^2^ would be established during an *in vitro* ALI exposure.^62^ Using the given experimental conditions and 100 nm SiO_2_ particles, they calculated an exposure period of about 400 hours would be needed.^16^ Thus, comparisons of most *in vitro* studies using submerged cultures and extremely high doses of particles with ALI experiments without enhanced deposition efficiency are not possible. Yet, *in vivo* on inhalation particle load is significantly lower and therefore much more similar to the ALI methods.^2^

**FIG. 2.**
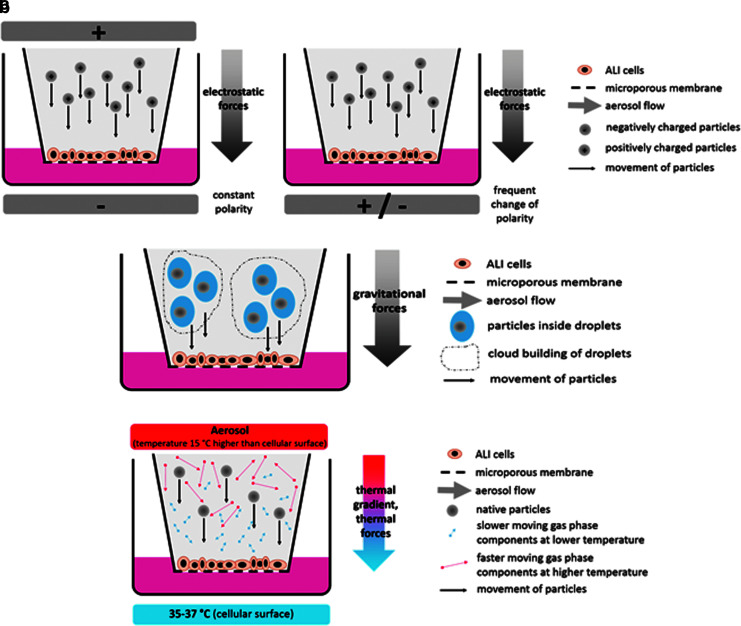
Main methods to improve particle deposition. **(a)** Electrostatic deposition on ALI cells to enhance particle deposition from aerosols. Aerosol particles are charged unipolar (left) or bipolar (right) and forced onto the cellular surface in an electric field with a constant (unipolar setup) or frequently changed (bipolar setup) polarity. **(b)** Droplet deposition on ALI cells. Particles are suspended in aerosol droplets to increase the particle size. Aerosol droplets are deposited on the cellular surface by gravitational forces. Depending on experimental conditions, this procedure may be enhanced by cloud movement effects of highly concentrated aerosols. **(c)** Enhancement of native particle deposition on ALI cells by thermophoresis. A thermal gradient of 15°C–20°C is applied between the cells and the aerosol, leading to a higher velocity of molecules in the aerosol gas at the warmer side. By this thermophoretic effect, aerosol particles are forced to the cellular surfaces of ALI cells.

Experimental approaches to improve this situation are mainly based on three principles: (1) electrostatic deposition, (2) droplet deposition, or (3) thermophoresis (also known as thermal precipitation) (Fig. 2). Electrostatic deposition involves the biological test system being exposed during alignment in an electrical field of usually 1000 V or higher.^63–65^ Using a unipolar setup, the aerosol is positively or negatively charged and the aerosol particles are forced to the surface of the exposed cells by electrostatic forces. Also, uncharged aerosols have been used for this strategy by using the natural charge of aerosols.^66^ In a modification of this basic electrostatic approach, bimodal charging of the aerosol has been introduced in combination with a continuously changing polarity of the electrical field around the exposed cells.^67,68^ Following this approach, a more balanced deposition of positively and negatively charged particles was pursued with the aim being an electrically neutral result in terms of the exposure process. The main advantage of setups based on electrostatic deposition is high deposition yields, which in theory can reach up to 100%. However, due to practical limitations and adaption of the physical principle to biological exposures, deposition rates reported for these systems were between 4% (130 nm silicon dioxide^66^) and 47% (500 nm polystyrene latex spheres^63^). Common concerns regarding electrostatic deposition strategies include possible adverse effects on the exposed cells due to high electrical fields and the separation of electrical charge of ionic components in culture media may also be an issue that has to be evaluated. For example, in A549 lung epithelial cells at a voltage of 1 kV, no adverse effects were observed using a basic (but insensitive) parameter for cell toxicity (lactate dehydrogenase release).^7^ Clearly, more cell systems need to be addressed in the future to draw firm conclusions on the effects of strong electrical fields. Also, under debate are possible effects of changes introduced to aerosol particles through electrical charging and the consequent changes in biological effects that might result. For example, it was observed that especially with respect to smaller particles, a change of electrical properties of particles might change their behavior in cellular uptake and toxicology.^69,70^

As an alternative to electrical deposition setups, droplet deposition has previously been reported.^6,71,72^ Here the test material is suspended or dissolved in a liquid medium and applied as a droplet aerosol in a small chamber through nebulization (via a commonly used nebulizer system; Aeroneb^®^; Aerogen). The cloud movement effect resulted in a highly effective deposition rate of about 56%. However, this approach cannot be applied to gases, sampling aerosols, or testing particles maintaining their dry physicochemical characteristics. Being based on droplet application, it has a clearly different objective than the other approaches for improvement of particle deposition from aerosols during ALI exposure.

The most recently applied approach for particle deposition improvements involves the thermophoretic effect (also known as thermal precipitation). In an unusual alignment of cells cultured on the bottom side of culture inserts, a setup has been established^73^ where particles are forced from horizontal aerosol flows onto the surface of cells during exposure. The thermal precipitation approach has some fundamental advantages. First, by principle, the exposed biological material should not be affected by the physical effect used, since a thermal gradient is used with the standard culturing temperature of the cells (35°C–37°C) on its colder side. Second, thermal gradients applied in the system were in the range of 15°C–20°C higher than the cell culture temperature. Thus, nearly no pretreatment of the test aerosol is needed, which makes it very applicable from the practical perspective and since the temperature of the aerosol can be applied very shortly before contacting the cells, no significant changes in physicochemical properties of the aerosol are expected.

Thermophoresis was also used in a further development where it was combined with a stagnation flow arrangement and a consequent use of consumable standard microplates throughout the exposure experiment.^40^ As a result, this exposure system does not require any change of the cellular environment before or after single or repeated exposure and offers multiple test groups on one plate in a very compact design. Moreover, it has completely separated compartments for exposure atmosphere flow and culture media. Hence, only the relevant route of exposure from the test atmosphere toward the cells is enabled, whereas any second route of exposure via the contact of the exposure atmosphere to the culture media is completely prevented.

### Overview of cellular models used at the ALI

For the purposes of testing inhalation toxicology endpoints, animal-based studies are limited in a variety of ways. These include ethical questions relating to animal experiments, their high costs, and questions about their biological relevance as rodent and human airways are anatomically very different.^74^ This particular aspect means the resulting air flow rates and air speed in the airways of animals differ from those of humans and this means there will likely be differences in the deposition of particles. Taken together, alternative methods that can replace, refine, and reduce animal-based experiments are very much needed, not least from the perspective of better representing biological processes that actually happen in humans. This is despite many regulatory assessments currently requiring animal testing in various domains.

#### Relevance to the *in vivo* situation

The trachea of a mouse has an internal diameter of ∼1.5 mm and is lined by a pseudostratified epithelium with about 55% ciliated cells, 30% basal cells, some secretory cells, and sparse neuroendocrine cells. In the rat trachea there are more ciliated cells in the epithelium.^75^ In the mouse, there are six to eight generations of intrapulmonar branches with a stereotypical branching pattern.^76^ The terminal bronchioles leading into the bronchioalveolar duct have less ciliated cells (∼26%) compared with more proximal airways. In contrast, the average human trachea has an internal diameter of ∼12 mm. There are more generations of intrapulmonary branches than in the mouse, and cartilage plates and smooth muscle surround the intrapulmonary airways deep into the lung. A pseudostratified epithelium built with basal cells and ciliated and secretory (mucus-producing goblet cells and club cells) cells lines these airways, whereas intrapulmonary airways in mice are characterized by the abundance of club cells.

In terms of anatomical features of the respiratory tract, potential regions of interest include the nasal and oral cavities, larynx, trachea, and alveolar region and commercial solutions to model these are already available for all these regions except for the alveolar region.

A key requirement for any alternative method is the relevance and comparability with the anatomical feature being modeled. For respiratory health, this means relevance in relation to the respiratory tract in humans. As already mentioned, there are key differences in the respiratory anatomy of humans and mice/rats, which means interpreting data from cell models derived from rodents in the human context is fraught with difficulty. A key principle here therefore is that, if possible, human-derived cells should be used for modeling human-related respiratory issues and that rodent based-cells should be avoided.

ALI systems therefore have the potential to conceptually provide relevant data since it is possible to construct them from human-derived cells and create modeling scenarios that are close to what might occur *in vivo*. For example, dosing can be exactly controlled and measured and particles or aerosols are not altered by contact with cell culture as would happen in a submerged model. Therefore, exposure of cells at the ALI is highly relevant for the physiological *in vivo* situation. In comparison to *in vivo* modeling with animals, dosing with ALI systems can also be controlled in much more accurate ways.

#### Respiratory cellular models

**FIG. 3.**
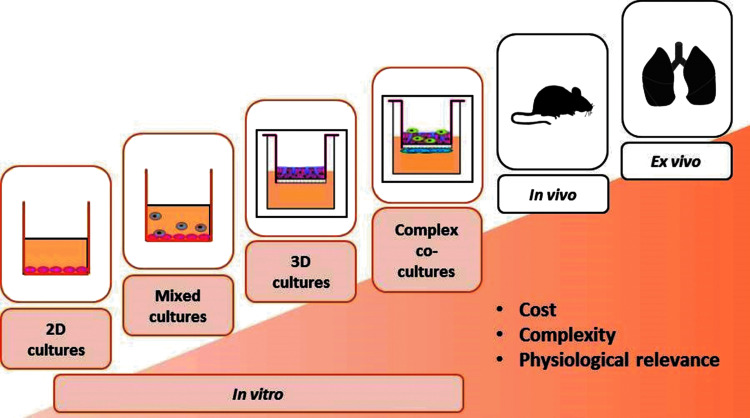
Respiratory models available from simple to complex. Costs and also physiological relevance increase in line with the complexity of models.

In terms of pulmonary models, a wide choice is available in terms of complexity (Fig. 3). The standard submerged two-dimensional cell culture is a simple procedure that is low cost and amenable to high-throughput processing. The complexity of *in vitro* models can then increase with the addition of characteristics that increasingly match the reality seen in humans. Examples of such models include mixed cultures (i.e., two or more cell types in one well) and three-dimensional (3D) cultures. Options do exist for using rodents and finally even human *ex vivo* tissue slices. While costs increase in line with the complexity of models, the physiological relevance also increases and this means the quality of data obtainable from more complex models is significantly improved.

The standard submerged cell culture cultivates the cells submerged in culture medium, which means it does not reflect the reality of the lung where cells are exposed to air. This means relevant cell culture modeling relating to the lungs and respiratory tract usually require an ALI if they are to have any relevance to reality in humans.

The development of ALI has been driven by groups interested in the biological effects of particles, as particles and nanoparticles react with proteins and/or lipids present in cell culture medium as this is the case for submerged cultures. This means that the biological activities of (nano) particles may change^77,78^ resulting in a corona formation, which in turn results in changed toxicity and altered cellular uptake. In short, a liquid interface will likely result in effects that in reality do not occur when cells from the pulmonary tract are placed in their more biologically relevant environment—namely at the ALI. One has, however, to keep in mind that the lung cells are covered with an aqueous lining and a mucus or surfactant lipid layer, which also can result in a protein corona formation of (nano)particles.^79^

Many relevant cell types have been investigated in combination with ALI. This has included primary cells,^80^ single cell line cultures,^81^ 3D,^82,83^ and cocultures^84^ tissue slices,^85^ and recently, several publications have emerged that have used organ-on-a-chip approaches.^86^ Considering primary cells, inter- and intra-individual variation can be considerable^87^ in terms of donor source.

The application of an ALI in cell culture models is widespread according to published literature. Examples include the use of skin cells,^88^ corneal cells from the eye,^88^ cells from the gingival epithelial mucosa,^89^ or various cell types from the respiratory tract,^90–92^ and also combinations of cell types (e.g., skin with melanocytes, epithelial lung cells, and immune cells).^10,93^ Sources of cells have included human, chicken, and rodents.^88^ Applications, meanwhile, have included the effects of (nano)particles,^93^ viruses,^94^ and bacteria,^95^ and also various chemicals,^96^ pharmaceuticals,^97^ and tobacco products.^82,89,98^

Many cell types from the pulmonary system have been used in combination with an ALI. For the epithelial cells of the airways, there are a number of cell lines available. These include Calu-3,^99–101^ 16HBE14o,^102–104^ and BEAS-2B.^105–108^ Alveolar epithelial cells can be modeled with A549 cells although they miss functioning tight junctions.^109–111^ Immortalized human alveolar type 2 cells with alveolar type 1 phenotype can also be used as a model.^112^

In terms of 3D cultures, there are many examples of possible models for the respiratory tract. These include primary endothelial and epithelial cells,^113,114^ a triple cell coculture model (comprising epithelial cells, primary macrophages, and dendritic cells^115–117^), and a tetraculture model (comprising epithelial and endothelial cells, macrophages, and mast cells^118,119^). Commercial solutions are also available that model 3D human reconstructed bronchial airways (Epithelix and MatTek are examples). Airway cells are obtained from patients undergoing surgical nasal polypectomy or lung lobectomy. The epithelial cells are isolated by enzymatic digestion. After amplification, the cells are seeded directly on the microporous membrane of Transwell inserts (e.g., 24-well format, Cat. No. 3470; Corning) and grown in a commercially available defined airway culture medium (e.g., EP04MM from Epithelix). Once confluent, the cultures are switched to ALI. After 3–4 weeks of culture, the epithelium becomes fully differentiated. As mentioned earlier, an organ-on-a-chip approach has also been developed, including several cell types.^120^

A large *in vitro* toolbox is available from which specific biological test systems can be set up. It includes cell lines, primary cells,^121^ or reconstructed 3D models, and primary *ex vivo* tissue such as precision-cut lung slices^41^ from different species and different regions of the lung.

Cell systems (both single- and multi-cell cultures) and tissue slices exposed to ALI conditions may also produce surfactant as shown for A549 cells exposed to air,^122^ which is protective for the cells and will interact with the particles before contact with the cells underneath.

Comparisons between conventional and ALI cell cultures show considerable differences in terms of biological readout. For example, messenger RNA (mRNA) expression of a variety of inflammatory mediators is considerably blunted when A549 cells are submerged and exposed to ZnO at the same concentration.^123^ Likewise, the release of IL-8 from A549 cells is considerably higher when exposed to Aerosil200 and SiO_2_ and submerged than when exposed via an ALI.^7^ More recently, a coculture of A549 and THP-1 cells showed higher sensitivity to poorly soluble nanomaterials (TiO_2_ and CeO_2_) when exposed at the ALI compared to submerged exposure.^8^ Loret et al. also highlighted the importance of considering the deposition rates when comparing ALI to submerged exposure.

All exposure and culture models described so far operate under ambient air pressure. However, this is not the case in reality where air pressure varies according to breathing rate and intensity. Although not high throughput, yet a system does exist that allows the culture of alveolar cells under breathing movement conditions.^124^ Recently, an organ-on-a-chip solution was presented where the pressure can be altered during culturing.^86^

### Conclusion

ALI exposure systems, in whatever format, resemble *in vivo* exposure conditions in a much more relevant way than standard approaches to cell culture. However, there are many factors to consider ensuring experimental conditions remain relevant to what is likely to happen in reality. While testing of pure gases is promising in well-established basic exposure settings, the testing of aerosols requires more extended experimental methods, involving also additional physically engineered developments as a basis for relevance and practicability. Cellular characteristics of the biological material such as metabolic capacities, surfactant or mucus production, tight junctions, and many other features are also important to consider. Especially for routine testing, commercial availability and costs may be issues. As a result, the biological relevance of ALI models for testing of inhalable effects of airborne material seems currently unbeaten.

Experimental data also show that there are clear differences between observed effects when cells are exposed via submerged conditions as opposed to ALI exposure. Indeed, culture medium will react with particles and alter their physicochemical and hence toxicological properties. Finally, dose is highly controllable and measurable under ALI and proper information on applied doses is important for understanding and interpreting any observed biological responses. In short, ALI systems are more relevant to the *in vivo* situation than any other currently available *in vitro* approach based on submerged cell cultures.

## Short Oral Presentations and Poster Session: Recent ALI Research Developments

A series of short presentations and a poster session followed the plenary lectures. They highlighted the latest progress being made with ALI *in vitro* inhalation models.

Presentations covered topics such as the use of lung surfactant to predict acute lung toxicity of inhaled chemicals, the comparison of ALI versus submerged exposures, the use of organotypic EpiOral^™^ tissue cultures as a model for inhalation studies, the presentation of new human *in vitro* 3D ALI models for small airways and lung cancer, the use of 3D ALI *in vitro* lung model for assessing repeated exposure to nanoparticles, and the use of ALI models to study effects of cigarette smoke and diesel exhaust exposures on asthma and pulmonary disease(s).

During the poster session, a number of themes emerged, including new automated approaches and the assessment of effects of various chemicals (printer ink, hair-straightening products, carbon nanotubes, waterproofing products, pharmaceuticals, and e-cigarette vapors), using ALI approaches. Methods to assess long-term exposures and low-dose exposures were also reported.

Brief summaries of the poster and oral presentations are given in Supplementary Data.

## Validation: Expert Panel Discussion

While methods and core principles relating to ALI approaches are clearly developing, it is widely accepted in the community of users that the approach is, at least in theory, superior to submerged culturing of relevant cell types. Key questions remain, however, in relation to validation.

At present, no *in vitro* methods relating to inhalation toxicology are validated from a regulatory perspective and, in particular, no methods that use ALI approaches are validated. This is despite ongoing work to develop the methods further.

A core aim of the workshop was to discuss this issue, to assess what might be required in terms of validation and to put in place a series of recommendations to start a process of validation. From the outset it was recognized that validation of methods is important, but precisely what should be validated, how validation should proceed, and who should actually do the validating were key questions the participants at the workshop hoped to start answering.

### Overview of the validation process: the EURL ECVAM protocol

For those interested in a full description of the validation process and subsequent discussions, please refer to the Supplementary Data relating to this topic.

The process of validation usually refers to the process of taking a method from the research and development phase, through to the transfer of the method to application (this is widely called prevalidation), maybe in industry or wider research groups, and then to formal validation that involves many different laboratories that essentially aim to reach the same result regarding a set of test compounds or outcomes.

Initially, prevalidation might involve simple repetition of experiments and comparison of results. However, formal validation may go much further and involve 10–100s of groups of researchers all formally working to a set protocol and subsequent full statistical analyses of the outcomes.

EURL ECVAM is the central EU organization coordinating validation of alternative methods to animal research. At EURL ECVAM, the process for validation is divided into four stages. There is an initial assessment of a submitted method, actual validation studies, independent peer review, and finally, the publication of an EURL ECVAM recommendation on the validity of a method. This then may form the input for the development of an OECD test guideline relating to the method. Once that has been issued, it may be the case that a method can then be used for regulatory purposes. A final step may also include the development of an International Standards Organization (ISO) standard.

The initial assessment of a method by EURL ECVAM revolves around three criteria. These are (1) the scientific and technical aspects of a method, (2) the regulatory relevance of a method, and (3) the impact a method may have on 3Rs.

If a method satisfies the detailed criteria, it may then proceed to a validation study (it may not proceed to this stage if either the method is judged to be incomplete in terms of the criteria or alternatively that sufficient information/data on validity were submitted initially and that the method can already be judged as valid).

The actual form of the validation study will depend on the level of validation already achieved. Following the completion of the studies, an independent peer review is carried out by EURL ECVAM's Scientific Advisory Committee (ESAC) who then issue an opinion. This forms the basis of the final EURL ECVAM recommendation. The final stage of validation is international recognition and regulatory acceptance, which can take many forms, including the development of OECD test guidelines and ISO standards.

In conclusion, the overall message in relation to validation is that ALI approaches for inhalation toxicology *per se* can be put through a process of validation if the steps outlined are followed and the time and budgets available to actually do it.

### Validation of ALI techniques: challenges to overcome

While the theoretical and regulatory perspectives of validation illustrate the usual issues facing teams wishing to validate methods (in particular, quite why validation is a laborious process), a perspective emerged that perhaps starts to illustrate a different issue that ALI *in vitro* modeling technology may face—the question of what to validate.

The setting for validation studies might vary widely because different user groups have different endpoint aims. From a regulatory perspective, a method should be able to predict likely endpoints that are relevant to human health (and particularly safety, from a regulatory point of view).

In that broad sense, there is a need to demonstrate that a method delivers in terms of relevance, applicability, repeatability, reproducibility, transferability, and predictive capacity. From a regulatory perspective that might well make sense and indeed that is the overall method that is used to validate methods.

However, from a research perspective, the case is different. The key aim here, in theory, is that an *in vitro* method represents the human *in vivo* situation as closely as possible. The conclusions of experiments using *in vitro* methods should after all be representative of what happens in humans and be predictive of outcomes.

Unfortunately that is not the case. In many cases, as was demonstrated at the workshop, the level of validation with respect to inhalation toxicology methods and particularly ALI approaches was small to nonexistent. Particularly from the perspective of research, the level of validation of methods was minimal, as reported by the participants at the workshop.

From the discussions (which are extensively reported in the Supplementary Data), several key points have been raised by the experts to consider regarding the validation of ALI methods for mimicking inhalation toxicology endpoints.

The first issue that was pointed out is that at the present time, nobody knows what models to develop in terms of validation and even whether it is realistic to expect that a limited number of models can ever adequately provide solutions for inhalation toxicology endpoints. In other words, the basic question is “validation is fine and needed, but what should be validated?” Considering the sheer number of endpoints, it seems unrealistic to expect one model would ever be sufficient for either regulatory assessments or research and that it was more likely that a series of methods would be needed. These might focus on specific local and systemic effects or on specific endpoints such as irritation, inflammation, carcinogenesis, fibrosis, or sensitization. There is a pressing need to catalog currently available methods and to then ask what is needed soon to answer the most pressing research questions. There is currently no real toolbox of approaches available and therefore nothing to actually validate.

Key points

- Numerous endpoints means it is unrealistic to expect one or a small number of methods will be sufficient to adequately assess all inhalation toxicology endpoints.
- No catalog available of research methods currently and no real list of the most pressing research questions make deciding on what to validate hard to answer.
- Agreement on a widely accepted standard to benchmark methods could be a useful tool for the community.

The second highlighted issue was a critical lack of standardization between groups in the field, which means comparing experimental outcomes is often difficult. There are still many open questions relating to these systems and approaches, not least from the perspective of validation. In a regulatory perspective, a wider demand for validated inhalation toxicology *in vitro* methods was highlighted with the general points being that the area is of high interest for governments and that in fact new EU legislation will mean much better methods will be needed for assessing a variety of endpoints in the near future.

Key points

- Lack of validation and standardization in the field makes comparing experimental outcomes difficult.
- Validation is one of many open questions relating to ALI systems.
- There is a general demand for relevant validated methods.

One of the more striking issues to arise in the discussions related to *in vivo* human relevance of methods. This issue was clearly highlighted suggesting that methods should ideally represent real human outcomes but that currently, reference points for inhalation toxicology are weak to nonexistent with little human data to go on. Certain approaches to improve this situation were proposed, including the use of epidemiological data possibly from occupational environments.

The fact was highlighted that some *in vitro* methods are only benchmarked against animal data and, in certain cases, these data are historic and of questionable quality given new data that have since emerged.

Key points

- Human relevance of models is essential, with validation taking place against human data.
- Little human data available to actually benchmark validation outcomes.
- Historic (animal) data may be of questionable quality.

## Recommendations

While it was clear that validation of inhalation toxicology methods and especially ALI models face many hurdles, a number of suggestions did emerge on a possible route forward. As mentioned already, standardization of approaches should be a key step to address many of the issues raised above. In particular, it was suggested that, however, many methods are needed to assess the endpoints desired, it was crucial that they are comparable between users. However, agreeing on what to validate remained a key issue. Especially for regulators, it appeared important and much needed to set priorities as a first step. A number speculated that because of the diversity of methods available it was possible that there are simply too many options available for anyone to agree on where to start. It may also be the case that the field has developed in a fragmented manner and simply that validation has not been a priority (yet) of many of the researchers actively developing methods. However, given the evidence of clear lines of demand for validation in the field, it was recognized that it was now important to get started on the process as a priority.

**FIG. 4.**
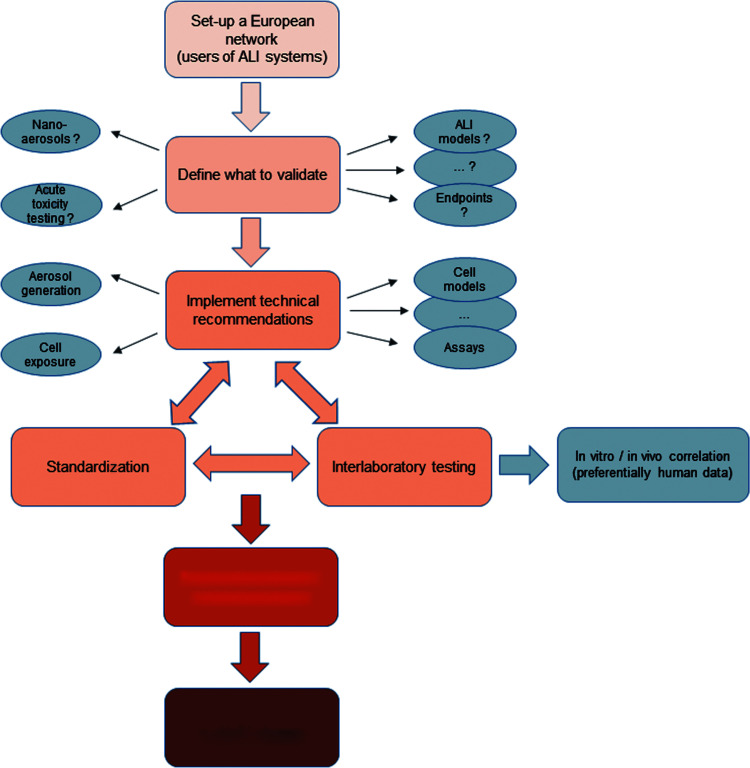
Initiation of the validation process for the use of ALI systems in the field of respiratory toxicology. EURL ECVAM, European Union Reference Laboratory for alternatives to animal testing.

To push ahead in this direction, some recommendations have emerged during the discussions (Fig. 4).

### Strategic recommendations

Considering the clear hurdles in starting such a process, the first recommendation is to organize a network to define strategy, combine previously available information, and to define and execute trials to start the validation process. It is recommended to:

(1) Set up a dissemination strategy, including website, for exchange of methods and technology,
(2) organize a specific scientific event such as a workshop or meeting on a regular basis,
(3) categorize diverse methods and topics to define an initial direction of validation [such as “acute toxicity testing” or “(nano)-aerosols” or “efficacy testing in pharmacology”], and
(4) implement technical recommendations such as those below to define a first common level of “good *in vitro* inhalation praxis.”

### Technical recommendations

One future challenge will be to find an appropriate strategy to guide the diversity of methods and applications into the direction of harmonization and standardization. This involves the biological test system, the exposure design, and the choice and implementation of cellular readouts at the same level of importance.

With regard to the exposure design, two principle ways may be present. One way may be the definition of a single validated setup involving procedures and equipment for generation and characterization of exposure atmospheres and exposures of a limited number of biological test systems. The other way could be a definition of process parameters for any setup to be defined and used in such an approach. Since the latter represents a more open harmonization and standardization strategy applicable to the constantly growing number of new applications and strategies, this would result in a living and growing number of documents that would have a higher acceptance from users. Briefly, it would include the definition of process parameters such as quality, performance, and validation criteria for all components of the *in vitro* experimental design. This would include the following:

(1) The generation and characterization of the test atmospheres.
(2) The characterization of the cellular exposure, the biological test system, and the readouts applied.
(3) And, last but not least, a clear documentation of all experimental parameters and outcomes (with respect to test atmospheres, exposure, biological test system, and readouts).

On the one hand, the focus of these criteria would be technical performance criteria (i.e., technical/physical/chemical criteria), but also biological performance criteria that are strongly related to individual applications are necessary.

They should include the following:

(4) Clearly defined control situations (positive and negative controls), which in the best case should be selected based on human data to establish a good control in the direction of translation from the *in vivo* to the *in vitro* situation.

Such a strategy for harmonization/standardization in the field of cell-based *in vitro* inhalation sciences could be the basis for further development, as it is urgently needed as an alternative method also in the sense of the 3Rs.

### Scientific recommendations

ALI methods are for many reasons considered to be more advanced than classic (i.e., submerged) *in vitro* exposure methods. Nevertheless, the final point for validation of ALI methodologies will be to define clear situations where ALI methods should be used instead or in support of animal experimentations or classic *in vitro* methods. This is a prerequisite for the use of ALI methods in a regulatory purpose. Defining clear situations would require the following:

(a) Comparison studies between ALI and animal experimentation and/or human data if available.
(b) Comparison studies between ALI methods and classic *in vitro* methods.

Some comparison studies have been performed recently, between ALI and classic submerged experiments. Nevertheless, few *in vitro* comparisons data are available. *In vivo*, no clear comparison data with ALI are available yet in the literature, although some comparison studies have been performed between *in vivo* and *in vitro* using classic submerged conditions. At this moment, it is thus not yet possible to define clearly when ALI methods should be recommended and there is an urgent need for more comparison studies, in particular between the “ALI” and the *in vivo*.

To generate reliable comparison data between *in vivo*/ALI/classic *in vitro*, it is recommended:

(1) To use cell models representative of the target tissue *in vivo.*
(2) To measure the exposure doses and to use relevant and comparable dose metrics.
(3) To use similar endpoints.
(4) To perform similar statistical comparisons.

## Conclusion

The development of ALI approaches to assess inhalation toxicology endpoints continues apace. Many of these advances were highlighted in the workshop and there was a general expectation that developments will continue, particularly in collaborative research projects. It was recognized by all that the significance and relevance of data from ALI strategies, although not validated until now, are expected to be a key strategy for establishment of alternative methods in inhalation toxicology and research.

However, it became clear that key questions remain in relation to a number of issues and particularly validation. Demand for validated methods remains high among academic, industrial, and regulatory organizations, although for strikingly different reasons.

The numerous endpoints that can be tested in inhalation toxicology mean that a considerable diversity of methods has emerged. When considered in the light of validation, the seemingly simple question of what to validate emerged as a key concern of many workshop participants. The relevance of methods, particularly in relation to reality in humans, was also highlighted as an issue that should be addressed particularly as new methods develop. While many agreed on the necessity of validation, the process was not immediately apparent. To assist the process, a general introduction to validation was given by speakers with experience in the area. Through this it became clear that priority setting was needed and subsequently that a network be established to achieve this and to subsequently design and run validation studies in the area.

To conclude, the workshop brought together key experts and users of ALI models to discuss developments in the area, and consider the question of how to start a process of validation. Recommendations included the establishment of a network to ensure validation commences in the area.

## Supplementary Material

Supplemental data
