# Supplementary material for: Air–Liquid Interface In Vitro Models for Respiratory Toxicology Research: Consensus Workshop and Recommendations
Source: Appl In Vitro Toxicol. 2018 Jun 1;4(2):91–106. doi: 10.1089/aivt.2017.0034 (PMC7500038; doi:10.1089/aivt.2017.0034)
Supplement: Supplemental data [file Supp_Data.pdf]

Supplementary Data

AIR–LIQUID INTERFACE WORKSHOP

Short Oral Presentations: Recent Air–Liquid Interface Research Developments

A series of short presentations followed the plenary lectures. They highlighted the latest progress being made with air–liquid interface (ALI) *in vitro* inhalation models.

Use of ALI of lung surfactant to predict acute lung toxicity of inhaled chemicals

Dr. Søren Thor Larsen of the National Research Center for the Working Environment, Copenhagen, Denmark, highlighted an ALI assay comprising lung surfactant and its use as a tool to predict acute lung toxicity.

Inhaled chemicals, including particles, may be able to reach and settle in the alveolar level of the airways. On settling, these particles first encounter the lung surfactant, which is located at the ALI in the alveoli and terminal bronchioles. The lung surfactant is a complex mixture of surface active phospholipids and surfactant-associated proteins. Although also involved in immunological processes, the most important role of the lung surfactant is to reduce and control the surface tension of the alveolar lining during respiration.[1]

At the alveolar level, the inhaled chemicals may interact with components in the lung surfactant. Normally, the physiological consequence of this interaction is negligible, but occasionally, it may change the properties and function of the lung surfactant. In cases of a significant reduction in the lung surfactant function, this may compromise the lung function and lead to acute lung injury or even acute respiratory distress syndrome.

One group of chemicals that are frequently causing acute lung injury in humans is the waterproofing spray products, which are used to achieve water-repellent surfaces on, for example, shoes, textiles, and building materials.[2] The toxic effect of waterproofing spray products seems to involve the lung surfactant, but little is known about what mixture of chemicals causes the acute lung injury. Against this background, an *in vitro* method was developed to screen chemical substances and consumer products for their acute lung toxicity.

To achieve this, a broad variety of waterproofing spray products were investigated by means of a constrained drop surfactometer.[1] Briefly, this approach is useful for assessment of surface active components in an ALI under dynamic conditions. A modified version of the constrained drop surfactometer bioassay was used so a lung surfactant film could be exposed to aerosolized chemicals during simulated breathing.[3] Curosurf, a lung surfactant extract from pigs, was used in the investigation.

To compare the results obtained by the constrained drop surfactometer method to *in vivo* toxicity, the same waterproofing spray products were administered to mice as an aerosol. The breathing patterns of the mice were monitored by plethysmography, and assessments of lung toxicity were based on irreversible reduction in the tidal volume. It was previously shown that this reduction in tidal volume correlates with alveolar collapse (atelectasis) probably driven by lung surfactant inhibition.[4]

Dr. Larsen briefly presented some results of the experiments, highlighting that they have now tested a large number of waterproofing spray products in both *in vivo* and with the constrained drop surfactometer method. Accordingly, some of these products appeared to induce acute lung toxicity in the animals. All products that were reportedly toxic in mice did also inhibit the lung surfactant function *in vitro*, giving a sensitivity (true positive rate) of the constrained drop surfactometer method of 100%. Furthermore, the ability of the model to correctly identify nontoxic products was also reportedly very high, and only three nontoxic products were misclassified as toxic by the constrained drop surfactometer method, giving a specificity (true negative rate) of the model of 63% as described.[5]

According to Dr. Larsen, the data suggest that assessment of lung surfactant function may serve as a valuable endpoint and a good predictor for *in vivo* toxicity of waterproofing spray products. Apart from these consumer products, the constrained drop surfactometer method has also reportedly proven useful for assessment of other substances, including pharmaceutical excipients intended for inhalation medicine.[3] Concluding, Dr. Larsen suggested that the constrained drop surfactometer should be further validated by investigating the *in vivo–in vitro* correlations for other products and substances. Based on these studies, it should then be assessed whether the constrained drop surfactometer method has the potential to be adopted as a test guideline to reduce or replace certain types of acute lung toxicity studies in animals.

ALI versus submerged exposure of poorly soluble metallic nanomaterials in alveolar cells

The exposure route in cell models often proves critical in determining biological endpoints in cell cultures. Dr. Thomas Loret of INERIS, France, discussed a study that examined ALI versus submerged exposures to poorly soluble metallic nanomaterials in A549 mono- and cocultures and the effects in terms of a series of biological endpoints.

The broad aim of the study, according to Dr. Loret, was to assess whether it was possible to better simulate *in vivo* effects with more complex *in vitro* models. To achieve that the authors report a comparison of cells (either A549 epithelial cells alone or with THP-1 macrophages) exposed to aerosols of four different metallic nanomaterials (three times different-sized titanium dioxide particles and cerium oxide) either via ALI or the submerged route for 24 hours (final dose achieved in 3 and 24 hours). In parallel, they also have reportedly exposed rodents to the same conditions although did not report those results at the workshop. ALI exposure was achieved through a system that has been reported before.[6,7] The biological effects assessed included cytotoxicity, inflammation, and oxidative stress endpoints.

In terms of dose, they report that for the ALI route of exposure, they achieved a maximum deposition of around 3 $\mu g/cm^2$, corresponding to a deposition efficiency of 15%–20%. Accordingly, they report they could observe some significant effects of the nanomaterials, considered to be slightly toxic, but only when the ALI exposure route was used and only in the cocultures. They also observed a general pattern that the ALI system was more sensitive at lower doses, in comparison to the submerged method and that cocultures were more sensitive than monocultures. To illustrate the results, Dr. Loret discussed a number of specific endpoints, including outcomes for proinflammatory markers.

Taken together, they concluded that the ALI method seems to be more sensitive than using the classic submerged exposure route. Also, when ranking the different compounds according to toxic effects, the mode of exposure made no difference to outcomes. More details on this study can be found in Loret et al.[8] In terms of perspectives, the authors will now compare their data with that of the *in vivo* exposure experiments they reportedly ran in parallel and make an assessment of which exposure method is the most appropriate to use in comparison to *in vivo* outcomes.

Organotypic EpiOral tissue cultures as a model for inhalation studies

Dr. Filippo Zanetti of Philip Morris International R&D (PMI R&D), Switzerland, described the results of an interlaboratory comparison of MatTek's EpiOral™, a type of organotypic human epithelial oral culture model.

Development of *in vitro* tools that adequately mimic the *in vivo* interactions and mechanisms of action of toxic compounds is an important aspect of the 21st century toxicology testing strategy. Reconstructed human organotypic cultures are a promising model for their characteristics resembling native tissues [9–18] and the potential to reduce animal testing.

The possibility to expose organotypic cultures at the ALI makes them a valuable tool to study the exposure to different aerosols *in vitro.*[19–21] Recently, the use of gingival and buccal organotypic cultures has been also used,[16,22–24] through which the oral mucosa is of particular interest in the case of exposure to cigarette smoke, as the first tissue of contact following inhalation. While current publications describing the suitability of these models for ALI exposure response testing are still rare, researchers have shown that this model, for example, the reconstituted organotypic tissues of the oral cavity, expresses differentiated characteristics comparable to the *in vivo* situation.[12,13,16,18]

In vitro test systems, such as the organotypic buccal culture model EpiOral (MatTek Corporation, Ashland, MA), are becoming increasingly commercially available for use in consumer product testing as well as in inhalation toxicology for acute and repeat exposure response assessment.[16,25]

However, confirmation of the interlaboratory reproducibility of the response of the organotypic oral cultures to different stimuli is missing. The availability of standardized approaches on assay endpoints would be an important step for the validation of this model before wider acceptance in the field of inhalation toxicology. An interlaboratory reproducibility study among three laboratories was presented that focused on testing the response of MatTek EpiOral cultures to different treatments (control substances).

The laboratories aligned their testing protocols and investigated endpoints such as cell viability, inflammatory response, and xenobiotic metabolism of the organotypic cultures in three independent experiments. Cell viability was measured by assessing adenylate kinase release induced by Triton X-100. The same treatment was applied to measure rate changes of cell metabolism by an MTT assay. Samples were also tested for proinflammatory mediator (MMP-1, IP-10, IL8) release by enzyme-linked immunosorbent assay after stimulation with TNF-α/IL-1β. Finally, the activity of two cytochromes (CYP1A1/1B1), significantly represented in the buccal mucosa,[26–28] was measured after being activated by 2,3,7,8-tetrachlorodibenzo-p-dioxin and the expression of genes involved in stress response was also investigated by quantitative polymerase chain reaction.

The overall experimental results were encouraging for within-laboratory and between-laboratory reproducibility, thereby demonstrating that assays were transferable between the institutions.

According to Dr. Zanetti, this research is an essential step toward establishing standardized and validated assay protocols for the MatTek EpiOral cultures, contributing to advancement of alternative methods.

New human in vitro *three-dimensional ALI models for small airways and lung cancer*

The main function of the human airway epithelium is to generate a sterile atmosphere for the alveolar region where the gas exchange occurs. As a first line of defense against airborne pathogens, the airway epithelium acts as a key barrier through the use of mucociliary clearance and host defense mechanisms.[29]

Interest in the use of three-dimensional (3D) reconstituted human *in vitro* tissues has been increasing recently in terms of studying respiratory diseases such as asthma, chronic obstructive pulmonary disease (COPD), cystic fibrosis, and bacterial and viral infections.[30]

Dr. Samuel Constant of Epithelix Sarl, Geneva, Switzerland, reported on the establishment and characterization of a novel *in vitro* human small airway model called SmallAir™. According to Dr. Constant, primary epithelial cells were isolated from the distal lungs of humans by enzymatic digestion. After amplification, the cells were then seeded on microporous membranes of Transwell™ inserts. Once confluent, the cultures were switched to an ALI. After 3 weeks of culture, the epithelium became fully differentiated, with a morphology of columnar epithelium. Subsequent analyses showed that the epithelium was electrically tight and that the model also a highly expressed CC-10, a specific marker of Clara cells. As expected, few Muc5-Ac-positive cells (goblet cells) could be detected. The models also reportedly contain basal cells and ciliated cells that show ciliary beating and mucociliary clearance.[31] According to Dr. Constant, SmallAir can be used to further understand the origin and development of various respiratory diseases such as lung cancers.

Indeed, with more than 1 million deaths worldwide every year, lung cancer remains an area of unmet need.[32] Realistic human 3D models are required to improve prediction of outcomes in the preclinical stages of research. According to Dr. Constant, another model, OncoCilAir™ (from OncoTheis; www.oncotheis.com), may be of use in this regard.

Reportedly, it is a nonsmall-cell lung cancer *in vitro* model, which combines a functional reconstituted human airway epithelium, human lung fibroblasts, and lung adenocarcinoma cell lines.[33] And, according to Dr. Constant, they found that in this 3D microenvironment, tumor cells expanded by forming nodules, mimicking a human lung cancer feature. As a proof of concept, they tested the antitumor efficacy of a panel of investigational drugs, including selumetinib, trametinib, and erlotinib. Tumor growth measured by fluorescence confirmed that OncoCilAir cultures responded to anticancer drugs in a selective way, suggesting that they represent a predictive tool for anticancer drug evaluation. Following this, they also reportedly demonstrated that OncoCilAir may be used for translational testing of inhalation therapies.[33] In addition, repeated airborne delivery of compounds to OncoCilAir was achievable by nebulization, simulating the chronic treatment regimen using aerosolized drugs.[34]

According to Dr. Constant, these studies likely suggest that SmallAir- and OncoCilAir-based assays represent promising tools to provide new insights into this major area of lung diseases and might allow testing concurrently of the delivery efficacy and possible side effects of an aerosol therapy within a single culture.

Three-dimensional ALI in vitro *lung model for assessing repeated exposure to nanoparticles*

Over the past two decades, the advances in nanotechnology research have been concomitant with the overwhelming increase in engineered nanoparticle production for a diverse range of consumer, industrial, and biomedical applications, such as medicine, cosmetics, sporting equipment, or information technology.[35]

Human exposure to nanoparticles is likely to occur either at the working place (i.e., occupational exposure), through the use/disposal of consumer products, or by the intended nanoparticles' use in biomedicine. The possible portals for entry of nanoparticles into the human body are the skin, the respiratory tract via inhalation, the gastrointestinal tract via digestion, or the blood circulation via intravenous injection.[36] Nonetheless, inhalation is considered as the most important route of entry for aerosolized nanoparticles especially when considering occupational exposure.[37] Due to the inevitable human exposure, it is crucial to design reliable and realistic *in vitro* experimental strategies that can be used to gain an understanding on how nanoparticles could affect the respiratory system at the cellular level following the research principles of the 3R (refine, reduce, and replace animal experimentation).[38]

Savvina Chortarea, a PhD student in the group of Prof. Barbara Rothen-Rutishauser at the Adolphe Merkle Institute, University of Fribourg, Switzerland, described their institute's *in vitro* ALI lung model and how it has been used to assess acute and short-term repeated exposures to nanoparticles and its potential use in the area of risk assessment. The heart of the model is a triple-cell coculture consisting of a monolayer of human epithelial cells combined with the two most important immune cells in the lung, that is, primary macrophages and dendritic cells.[39] Depending on the anatomical location under investigation, the epithelial layer can consist of alveolar or bronchial cell lines or even primary cells.[40] Human blood monocyte-derived macrophages are then added on top of the epithelial cells and human blood monocyte-derived dendritic cells underneath the epithelial cells. The model can be cultured at the ALI and can be used with exposure systems such as the air–liquid interface cell exposure (ALICE) system.[41] The system has already been applied to assess acute/single doses of a variety of nanoparticles. Examples include round-shaped particles (such as gold,[42,43] silver,[44] and zinc oxide)[45,46] as well as fiber-shaped particles such as cellulose whiskers.[47,48] In all these cases, dosing was repeatable, dose dependent, and deposition was homogenous.

The system also allows repeated nanoparticle exposures to be performed. One recent example was carried out with carbon nanotubes,[49] where the 3D model of the human epithelial airway barrier, as described above, was exposed every 24 hours to doses of multiwalled carbon nanotubes that corresponded to human occupational exposure mimicking inhalation over several days or up to 5 weeks.[50] Carbon nanotubes were internalized by the cells, but there was, however, limited biological impact over the short-term exposure period. Further studies are ongoing to prolong the exposures and to also include healthy and diseased cells. While the study is ongoing, first results indicate no changes in morphology compared to negative controls, however, various biological endpoints associated with oxidative stress and inflammation do respond in comparison to negative controls.

By applying this approach it was possible to realistically mimic the inhalation of multiwalled carbon nanotubes by using the triple-cell coculture of the human lung epithelial tissue barrier together with the ALICE, providing an effective alternative to animal testing strategies. Furthermore, it was possible to investigate not only the potential acute toxicity of multiwalled carbon nanotubes but also to reveal the mechanisms that underlie the manifestation of potential adverse effects after repeated exposures and prolonged exposure times, demonstrating that repeated multiwalled carbon nanotube exposures have a limited biological impact on lung cell cultures at the ALI, over a 3-day period.

In conclusion, an advanced 3D multicellular tissue model cultured at the ALI in combination with a reliable air–liquid exposure system that mimics the inhalation of nanoparticles *in vitro* as realistically as possible is recommended for realistic risk assessment of inhaled nanoparticles.

ALI models to study effects of cigarette smoke and diesel exhaust exposures on asthma and pulmonary disease(s)

Dr. Pieter S. Hiemstra of Leiden University Medical Center, The Netherlands, highlighted the role for *in vitro* ALI models in disease research and particularly in asthma and COPD. With a focus on airway epithelial cell function, the research in particular is looking at inflammatory mediators, viral and antibacterial host defense, lipid mediators, and the resolution of inflammation and finally repair and differentiation in relation to respiratory diseases.[51] Interest in the disease relevance of stimuli means that for asthma there is a focus on Th2 cytokines,[52,53] while for both asthma and COPD, there is an interest in the effects of cigarette smoke[54,55] and diesel exhaust fumes.[56]

At the center of the research are a number of exposure systems that are used to study effects of such stimuli. The research on cigarette smoke exposure relied on a low-cost

modified hypoxic chamber with a ventilator with air as a control,[55] whereas for the diesel exhaust studies, a dedicated Vitrocell exposure unit was used.[56] Primary human airway epithelial cells are sourced from primary tissues such as tracheal tissue from transplant donors, bronchial tissue from resected lung tissue following cancer surgery and bronchial biopsies obtained during research bronchoscopy. Following protease treatment, cells are expanded, stored in liquid nitrogen, and finally, they are expanded and transferred into Transwell inserts. Differentiation then takes place at an ALI with the result being a range of differentiated cell types that resemble the airway epithelium *in situ* and that are amenable to experimentation.

Studies on acute exposure to cigarette smoke revealed increased stress responses and inflammation and decreases in host defence in cells (as determined by decreases in production of antimicrobial peptides such as human beta-defensin-2 [hBD-2] and decreased antibacterial defense). Increased expression of markers of oxidative stress (e.g., HMOX1) and endoplasmic reticulum stress/integrated stress response (e.g., GADD34) have also been observed. The studies have also revealed a dual role for airway basal cells in terms of lung repair and host defence, which is switched by cigarette exposure.[55]

Chronic smoke exposure studies for up to 3 weeks at the ALI have revealed that smoke can modulate epithelial differentiation and IL-13 responses. In ongoing studies, the combination of exposure to Th2 cytokines, the main driver of allergic airways inflammation in asthma, and cigarette smoke is being investigated.

In terms of research on diesel exhaust emissions, Dr. Hiemstra reported on collaboration with TNO (Dr. Ingeborg Kooter), The Netherlands, where the effects of exposure to whole diesel exhaust were investigated.[56–58] These studies revealed a partially similar set of effects from diesel exhaust as were observed with cigarette smoke. In particular, there was an increased oxidative stress response. There was also a decrease in inducible expression of the antimicrobial peptide BbD-2, and increases in integrated stress response and inflammation. This was all in the presence of limited cytotoxicity and limited effects on barrier function. According to Dr. Hiemstra, the studies demonstrate that *in vitro* exposures to cigarette smoke and diesel exhaust fumes have effects on relevant primary cells and that such experiments seem to prove useful in the realm of understanding adverse respiratory health effects.

Poster Session

Fifteen posters were presented at the ALI workshop to highlight a number of developments in the area of ALI approaches for inhalation toxicology.

(1) Toxicity evaluation of electronic cigarette vapors in human bronchial epithelial cells (Anthérieu et al., University of Lille, France). A study by Anthérieu et al. comparing e-cigarette vapors versus conventional cigarette vapors in an *in vitro* human bronchial cell culture smoking machine concluded that e-cigarette vapors may be less toxic than conventional vapors. Further studies are reportedly ongoing with other e-liquids (i.e., the liquids used in e-cigarettes) and in animal models to assess long-term exposure to e-cigarette vapors.

(2) Development of an *in vitro* inhalation toxicity test for improved protection of human health (Jackson et al., MatTek, Bratislava, Slovakia). The EpiAirway organotypic human airway model developed by MatTek is reportedly equal to current animal tests for predicting whether inhaled chemicals are highly toxic, at least according to a study by MatTek. Moreover, they claim the model is better than current animal tests at predicting whether inhaled substances are moderately/slightly toxic. They conclude the approach should provide improved protection of human health in comparison to currently used animal tests.

(3) Development of a repeated exposure protocol of human bronchial epithelial cells at ALI to study the effects of air pollution-derived fine particulate matter (PM) (Leclerq et al., University of Lille, France). Normal human bronchial cells and particularly diseased (COPD) cells can be cultured at an ALI and used in a repeated exposure scenario to assess a range of cellular endpoints after exposure to fine PM from air pollution, according to Leclerq et al. They conclude that the experimental strategy should allow underlying mechanisms of toxicity to be studied in more detail than before.

(4) Development of an *in vitro* test to assess the inhalation toxicity of nanomaterials (Sharma et al., PETA International Science Consortium Ltd, United Kingdom and others). Sharma et al. reported ongoing work on *in vitro* ALI approaches to assess various endpoints relating to the potential (inhalation) toxicity of multiwalled carbon nanotubes. Exposure of various relevant cell types to the nanotubes via a liquid interface indicated few endpoint responses. Comparative ALI studies with the reconstructed primary human alveolar tissue model, EpiAlveolar from MatTek, are reportedly ongoing.

(5) Nanoparticle exposure in air–liquid interface (ALI)—a more sensitive model for nanoparticle toxicity assessment compared to submerged exposure (Karlsson et al., Karolinska Institute, Stockholm, Sweden, and others). A straight comparison of cell exposure via liquid and ALI conditions again suggests that ALI exposure provides a more sensitive model for assessing toxicity of nanoparticles. In this case, exposure to silver and cerium oxide nanoparticles elicited no change in metabolic response in A549 cells when exposed via a liquid interface. In contrast, an equivalent exposure via an ALI resulted in reduced metabolic responses.

(6) A comparative long-term toxicity study of CeO_2 nanoparticles following standard monolayer culturing protocols and 3D complex airway epithelial models (Goñi de Cerio et al., Gaiker Technology Centre, Zamudio, Spain). Staying with experiments on the potential toxicity of cerium oxide nanoparticles, it appears that long-term exposure (3 months) does result in cellular damage in the Epithelix MucilAir model. Meanwhile, exposure to CeO_2 in classic monoculture experiments using A549 and Calu3 cultures had no effect on equivalent endpoints. In this case, they do not report whether these classic approaches used liquid or ALI exposures. They suggest their approach may be relevant for assessing the effects of chronic exposure to nanoparticles and therefore is particularly applicable in an occupational context.

(7) An experimental cell-based approach to determine biological effects of aerosols released during the use of consumer and cosmetic products *in vitro* (Ritter et al., Fraunhofer ITEM, Germany, and others). The cellular effects of exposure to hair straightener can now be assessed *in vitro* in a manner that mimics exposure that can be expected when the product is used by a consumer (when they spray it on their head to straighten hair). Specifically, Ritter et al., describe the experimental setup and its use to assess aerosol exposure in an acute toxicity design. Significantly, they report effects in comparison to both positive and negative controls. The hair treatment aerosols reportedly induced effects that were more similar to the negative rather than the positive controls. Given the relevance of the exposure scenario, they suggest further development might mean the approach can be used more generally in safety assessments.

(8) Three-dimensional airway models using the air exposure route: first steps toward an *in vivo* replacement (Kooter et al., TNO, The Netherlands). The ongoing development of the MucilAir 3D airway model of Epithelix by TNO is reported by Kooter et al., with a report on a set of comparison experiments between the 3D model and two monolayer cell cultures (A549 and BEAS-2B). Based on their data, they suggest their experiments indicate that the 3D model is more resistant to air and metallo-nanoparticles (cerium oxide and copper oxide) than the two-dimensional models and thus might be more predictive of *in vivo* effects. They highlight a number of parameters that should be considered when using the MucilAir 3D model.

(9) A laboratory-scale measurement technique for the air–liquid interface exposure of human lung cell cultures toward airborne nanoparticles (Mülhopt et al., Karlsruhe Institute of Technology, Germany, and others). Mülhopt et al. described the development and application of an automated cell exposure system, the Vitrocell Automated Exposure Station, operating in conjunction with ALI exposure that is designed to better mimic the actual processes that take place in the human lung. They described many of the controllable parameters of the system that should mean experiments and results are reliable. A series of experiments with cerium oxide using the system were also presented.

(10) A comparison of two air–liquid interface systems (Koekemoer et al., Utrecht University, The Netherlands). A comparison of the Vitrocell Automated Exposure Station and, another similar system, the Nano Aerosol Deposition Chamber for *In vitro* Toxicity was presented by Koekemoer et al. Both systems were tested simultaneously for deposition efficiency and cell viability following exposure to copper oxide particles. While there were some variations in outcomes, they suggest the systems both fulfil requirements for realistic *in vitro* testing but caution that further studies are needed to fully explain their data.

(11) Development of treatment strategies to perform repeated exposures of bronchial epithelial cells in air–liquid interface cultures to study the effects of particles (Boland et al., CNRS, Paris, France). Boland et al. reported that it is possible to culture normal human bronchial epithelial cells at an ALI and maintain them for several weeks, thus allowing long-term exposure experiments to be performed. However, depending on exposure scenarios, different phenotypes of cells developed. They suggest their approach means repeated dose exposures can be performed while maintaining integrity of cells and that meaningful metabolic endpoints can be studied.

(12) Cytotoxicity assessment of the emissions of a CdTe quantum dot-based fluorescent ink (Sánchez et al., Inkoa Sistemas, Erandio, Spain, and others). The cytotoxicity of inhaled printer ink containing cadmium telluride quantum dots is the subject of an ongoing study by Sánchez et al., with preliminary results suggesting that any toxicity is likely a function of cadmium concentration in the ink. BEAS-2B cells were reportedly exposed to the ink or standard solvent-based printer ink either via an ALI or in submerged conditions.

(13) Acute exposure of precision-cut lung slices to gaseous compounds and smoke-induced cytotoxicity and inflammation (Obernolte et al., Fraunhofer Institute of Toxicology and Experimental Medicine, Hannover, Germany). Precision-cut lung slices can be used as an *ex vivo* lung model according Obernolte et al. who reported on their use of the approach to assess the acute proinflammatory/toxic effects of a variety of gases and also cigarette smoke. They reported that the model could withstand exposure to ozone and nitrogen dioxide when cultured for an hour at an ALI. However, after 1 hour, there was a loss of viability and induction of cytokines. Similar effects were reported for cigarette smoke. They suggest that the model closely resembles *in vivo* exposure and so should be appropriate for toxicity testing.

(14) Airway epithelium cocultured with immune cells for a better assessment of the low-dose effects of environmental pollutants (Ricquebourg et al., Paris Descartes University, Paris, France). Coculturing the MucilAir 3D epithelial cell model with immune cells increases sensitivity to environmental pollutants according to Ricquebourg et al., which should now mean it is possible to study potential toxic effects of low doses of environmental pollutants. The study compared the effects of exposure of the model (cultured at an ALI) over 4 weeks to volatile organic compounds typically found in paint. When immune cells were included, the sensitivity of the model then increased.

(15) Lung surfactant inhibition as an indicator of acute inhalation toxicity (Da Silva et al., National Research Centre for the Working Environment, Copenhagen, Denmark). The use of a constrained drop surfactometer to measure the effect of particle deposition on lung surfactant was reported by Da Silva et al. Large differences in potency of different chemicals or particle types were reportedly observed. For example, albumin, which is known to cause surfactant

inhibition during lung damage, resulted in significant inhibition at low doses. Certain pharmaceuticals in contrast had no effect at all even at extreme doses. Meanwhile, chemicals used to impregnate surfaces for waterproofing had a variety of effects but significantly had a 100% detection rate when compared with known effects in mice. They suggest the method can be used to study any inhaled particle to get an indication of potential toxicity.

Validation: Expert Panel Discussion Transcript

The following is a transcript of the expert panel discussion on validation. Readers may find this content useful for the purposes of understanding the detailed discussions that occurred at the workshop.

Validation: the process

Dr. Philippe Hubert of INERIS, France, introduced the topic of validation of *in vitro* models and the many contexts in which the topic is set. According to Dr. Hubert, the discussion around the topic of validation usually centers on the step between validation and prevalidation, and on the perspective of acceptance of a method.

Three core questions relating to acceptance should be answered in relation to validation (or recognition): what are the needs of stakeholders, what tools are available for validation, and is there a role for prevalidation?

In terms of needs, these vary depending on perspectives. Usually, the view taken is that validation is part of a regulatory process. For industry, access to markets is key and to have that, one may require regulatory assessments and approval of products. To achieve that, there is of course a requirement for methods that can reliably predict risk. As such, any method designed to achieve this should be validated, otherwise, it is impossible to know whether a method is reliable. While this is a very practical perspective from industry, it is not the only one where validation is important.

Research and development also require methods that can reliably predict outcomes, otherwise making statements based on data from unreliable and nonvalidated methods becomes meaningless and conclusions cannot be drawn. Validation is therefore an important issue in toxicology and ecotoxicology, particularly where risk assessments are being undertaken. Equally, however, validation is important in biology, physiology, medicine, and so on. It is important therefore to realize that regulatory needs for validation are not the only needs and that many other areas have interests in validation.

In terms of regulations, it is often the case that methods validated by international bodies are the only accepted methods. However, some parts of legislation do allow the use of alternative ''recognized methods'' of assessing risk. An example of this is section 47 of REACH (registration, evaluation, authorization, and restriction of chemicals) regulations that explicitly state: ''In accordance with Directive 86/609/EEC, it is necessary to replace, reduce or refine testing on vertebrate animals. Implementation of this regulation should be based on the use of alternative test methods, suitable for the assessment of health and environmental hazards of chemicals, whenever possible. The use of animals should be avoided by recourse to alternative methods validated by the Commission or international bodies, or recognised by the Commission or the Agency as appropriate to meet the information requirements under this regulation.''

While the need is clear (implementation of 3Rs), in many ways, implementation of alternative methods is set in a narrow context to methods either validated by OECD or methods recognized by the European Commission (EC) but validated elsewhere. The legal basis on which the use of alternative methods is set is a so-called Weight-of-Evidence approach. This means that when there is sufficient evidence gathered from multiple sources or a validated method (either validated by or recognized by the EC) is available that is equivalent to an animal-based approach, the alternative method(s) should be used. In all cases, adequate and reliable documentation of methods and evidence is required.

There are many organizations in Europe involved in some way with validation of alternative methods. At the international level there is the OECD and International Standards Organisation (ISO), both involved in the standardization and validation of methods. At the European level there is the Joint Research Centre of the European Commission and specifically the European Union Reference Laboratory for alternatives to animal testing (EURL ECVAM) involved in validation and wider research into alternative methods. There are also European-level networks, PARERE and European Union (EU) NETVAL, involved in validation work. At the national level there are also many laboratories involved in various aspects of prevalidation or validation. Importantly, there are also many private initiatives involved in aspects of validation.

In terms of prevalidation, this can be seen as the step between the development of a method within a context of R&D and its first use outside of R&D. Examples here might include the use of a method within a start-up, scaling up of processes in industrial applications or in dissemination. It is a step that demonstrates some validity of the method, for example, in terms of development of a standard operating procedure and the demonstration of reproducibility or repeatability, but it is not the full validity required for a recommendation by EURL ECVAM or adoption by the OECD or ISO. According to Dr. Hubert, the step is a crucial one for the development of recognition of a method but that for many methods it is one that is not undertaken sufficiently or at all. This means many methods never make it beyond research applications even though there is potential for wider use both in terms of applied sciences and in regulatory contexts. The reasons for this are myriad, but likely come down to the process of validation being deeply unrewarding for the people doing it. In the context of research, a method may be developed for a very specific application and validated for that purpose.

However, to then validate it for further application may require a significant investment of both time and money—two resources that are limited in many cases. There may well be a case to invest heavily at this stage of development to ensure more methods progress to full validation. Once prevalidation has progressed sufficiently, the method and accompanying data may be used directly in applications (even as part of wider regulatory assessments) or as input for full validation and recognition by EURL ECVAM, OECD, and/or ISO.

In France, there are currently ongoing efforts to develop a prevalidation platform for endocrine disruptor assays and

methods. To illustrate the challenges involved with such an effort, Dr. Hubert highlighted a number of issues they have encountered in recent years.

The platform brings together organizations from the government, industry, retail, research, and start-ups and nongovernmental organizations (NGOs) and one of the biggest issues they have reportedly faced is governance. Specifically, managing conflicts of interests (whether perceived or real) and the makeup of the platform in terms of balance between public/private ownership and leadership have all been issues. Indeed, even deciding what methods to develop has proved challenging.

Financing, while always an issue to be resolved, required extensive efforts to put in place. For example, extensive discussion was needed to resolve whether upstream research organizations should pay for prevalidation or whether the end user should bear those costs. Experience shows that distribution of finance for facilities and studies can become extremely complicated and time-consuming. A specific issue highlighted by Dr. Hubert related to resolving intellectual property ownership and nondisclosure and that resolving such issues in a timely manner is an important step.

The current status of validation, both within the EU and globally, is that methods and their validation are constantly ongoing, with new methods emerging from ongoing research and a need to update methods that have been available for many years. There is also a need for the development of faster methods—the EPA is making considerable investments in this area—and these will of course require validation if they are ever going to be used in a regulatory context. Cost is always a consideration, and this means efforts are being made to also reduce costs and in the process validation will be needed.

Concluding, it is still an open question whether prevalidation is a necessary step toward full validation and indeed what prevalidation actually is, is still a point of debate for many. For example, definitions of prevalidation even vary at the level of international organizations. A possible advantage of prevalidation is that it may make the process of full validation faster since such data can be used as a starting point or input for organizations such as the OECD, ISO, and EURL ECVAM. Prevalidation data may also prove useful in regulatory frameworks that cover chemicals, foods, water, air, and so on. The primary purpose though for prevalidation should always be to make sure the methods are reliable and to provide some guarantees for nonregulatory purposes and research—in short, that data coming from a method can actually be used.

In terms of tasks for any organization (of any size) involved in (pre-)validation, tasks to consider may include an assessment of the relevance of candidate methods (possibly this may involve additional tests, handling data sets, identifying requirements of stakeholders, and even the precise identification of methods), to build up data for the validation process, to identify whether prevalidation adds value, and to work on dissemination to stakeholders associated with methods. Finally, funding for validation should be considered a crucial issue—validation studies can be very expensive in terms of time and money.

Dr. Hubert ended with a note on policy. The development and validation of alternative methods are becoming increasingly international, and ultimately, recognition of validation of these methods is made at a multinational level (i.e., at the level of the OECD and ISO). In that context is there still a meaning in having national centers dedicated to the development of alternative methods in an international system? This is a question that is often raised but remains the focus of debate to this day.

EURL ECVAM: an overview on method validation and acceptance

Dr. Laura Gribaldo from EURL ECVAM, JRC, Ispra, Italy, summarized the role of her organization in the development and validation of alternative methods and specifically the nature and workflow of the validation process of EURL ECVAM and regulatory acceptance.

EURL ECVAM was established under the Directive 2010/63/EU on the protection of animals for scientific purposes. Some of the responsibilities of the organization include guidance on research (normally applied research), coordination of validation studies, dissemination, facilitation of dialogue among stakeholders, regulators, and scientists, and finally, to promote international acceptance of newly developed methods. The process of validation happens in a number of steps. These include test method submission, validation studies, peer review, EURL ECVAM recommendations, and the promotion of regulatory acceptance of alternative test methods and approaches.

The validation workflow is divided into four steps. These are an assessment of a submitted method, validation studies, independent peer review, and finally EURL ECVAM recommendations on the validity of test. For the first step, the assessment of a submitted method, three criteria are used to initially assess a method. These are (1) the scientific and technical aspects of a method, (2) the regulatory relevance of a method, and (3) the impact a method might have on 3Rs—the replacement, refinement, and reduction of animal use in scientific experiments.

In this phase, the intention is to assess the completeness, accuracy, and quality of information provided on seven test method submission modules. These are test definition, within-laboratory reproducibility, transferability, between-laboratory reproducibility, predictive capacity, the applicability domain, and performance standards. For the test definition, the purpose of the test should be clearly stated, and the endpoint (s) for which it is designed to test should be described. Transferability refers to how robust a method is in terms of getting the same outcome in terms of sensitivity, specificity, and accuracy when performed in different laboratories. Predictive capacity is about the ability of the method to predict the endpoints measured and therefore it is the capacity to predict *in vivo* outcomes. The applicability domain is an important concept in that while the robustness of a method should be determined from an analytical point of view, its application to different fields of regulatory concern may (or may not) be wider than its original intended purpose. Taken together, the answers given in the different modules will define if and at what stage the test method will enter validation (or peer review).

Test method submissions can be made through the EURL ECVAM website. A technical guidance document is in preparation and this will specifically address test method submission to EURL ECVAM. The aim of that document is to

improve quality of submissions, since at the moment not all submissions meet initial requirements and are therefore rejected at very early stages. From time to time, EURL ECVAM also issues calls for test method submissions on specific topics.

The second step in the process is to undertake the validation study (if required). Using the example of the AR CALUX test method, which allows the detection of androgenic substances *in vitro*,[59] the validation study as a whole has taken the form of a multistage investigation scheduled to run over 4 years. In 2014, an initial selection of three EU test facilities was made and after collaboration agreements were signed, they worked up standard operating procedures and protocols and agreed on 52 chemicals as the subject of the validation. At the beginning of 2015, training was initiated in the three laboratories to ensure that the protocols were all carried out in a similar way. Following completion of the training period, the first validation study on transferability was carried out. In 2016, two further studies are planned in relation to reproducibility and predictive capacity. If those go according to plan, 2017 will see data evaluation and the writing of the validation report for eventual submission for peer review.

The independent peer review is carried out by ECVAM's Scientific Advisory Committee (ESAC), EURL ECVAM's independent scientific advisory committee. ESAC consists of 15 independent experts drawn from academia, industry, and government, and meets twice per year at the JRC in Ispra, Italy. The ESAC mandate is 3 years and was last renewed in 2013. Their primary role is to provide EURL ECVAM with independent high-quality scientific advice on the scientific validity of alternative test methods by performing peer reviews. The advice is provided in the form of ''ESAC Opinions'' that clearly specify the scientific rationale for the position taken.

After this peer review, EURL ECVAM issues a recommendation. This provides guidance on how a test can be used and may provide suggestions in terms of developing the test further. Recommendations that have been published to date are available on the EURL ECVAM website (https://eurl-ecvam.jrc.ec.europa.eu). While the workflow used remains the same for each submitted test method, it is worth stressing that each test method is evaluated on its merits and the process used, particularly in the validation study period, is customized to provide the information required for validation.

EURL ECVAM has other advisory/consultation bodies each with specific mandates and expertise. Regulatory relevance of test methods is an important aspect of validation, at least EURL ECVAM and PARERE provide advice on these aspects. The panel consists of member state representatives and relevant agency staff. Its role is to provide upstream input on potential regulatory relevance and suitability of proposed alternative approaches. It also has a role to identify approaches that may deserve attention from EURL ECVAM. These roles mean that EURL ECVAM can receive regulatory advice at the very beginning of any validation from the perspective of whether a test method is likely to have any regulatory relevance. This means EURL ECVAM can avoid costly evaluations of methods that are never likely to have any regulatory relevance. PARERE also provides input into EURL ECVAM strategy and draft recommendations following ESAC peer review.

ESTAF is a stakeholder forum that provides advice on test method relevance and EURL ECVAM strategy and recommendations. It comprised representatives from research, industry, and civil society organizations and is tasked with advising EURL ECVAM on test method validation from the perspective of end-users or other stakeholder perspectives. EURL ECVAM also has a number of global partnerships and networks that are designed to support the mission and mandate(s) of the organization. EU-NETVAL and ICATM are two examples.

The final stage of validation is international recognition and regulatory acceptance. Once a method is validated (both scientifically and from the perspective of regulatory relevance via EURL ECVAM), there are specific steps that can be taken to achieve international recognition and acceptance. Which steps are taken depend very much on the regulatory framework/context in which a method sits and the regulatory bodies that will ultimately be involved. Example instruments to consider include REACH,[60] Regulations for cosmetics,[61] International Council for Harmonisation of Technical Requirements for Registration of Pharmaceuticals for Human Use,[62] International Cooperation on Harmonisation of Technical Requirements for Registration of Veterinary Medicinal Products (VICH),[62] ISO,[62] (European) Pharmacopoeia,[63] or an application for an OECD test guideline.[64] In this respect, EURL ECVAM has a number of collaborations with the OECD panels involved in the development of test guidelines and that means they offer expert advice and perspectives on the test methods under evaluation. The recommendations of EURL ECVAM relating to the validation of alternative test methods are strongly noted by the OECD panels, according to Dr. Gribaldo.

In terms of the strategy of EURL ECVAM, these are defined in a number of different ways but are mainly focused on a range of different toxicological areas. The main drive of the organization is to address different regulatory areas and that means addressing issues in areas such as chemicals, cosmetics, pharmaceuticals, and biocides as examples. Specifically, this means identifying gaps and opportunities in relation to method development and validation and to identify gaps in specific areas where there is a need for such methods. Moreover, this means taking a focus on issues surrounding 3Rs and whether alternative methods are available or could be developed.

In terms of a scientific approach to validation, EURL ECVAM uses a concept called adverse outcome pathways (AOP). Broadly, this defines steps in a pathway that unravels the effects of a chemical in terms of its toxicological mode of action and how this relates to biological endpoints. This means that for EURL ECVAM, they use such knowledge, when discovered, to define so-called integrated prediction systems and that the pathways therefore are fit for the purposes of supporting safety decisions.

The chain of events that are tracked for the purposes of modeling the mode of action of a particular substance are as follows: exposure, the initiating event, the organelle, cellular, and tissue effects, the organ response and then the response in the individual, and finally, the likely effects at a population level.

In short, according to Dr. Gribaldo, this means they are ''facilitating a shift toward a knowledge-driven paradigm for chemical risk assessment.''

Validation in summary

For EURL ECVAM, it is not a question of if or why validation is necessary, but a question of how best to validate methods. A key message from Dr. Gribaldo was that when a method is submitted to them, a significant amount of their decision-making is based on whether (1) there is a clear mechanistic and biological description of the method, (2) the relevance in terms of accepted AOP knowledge is clear, (3) there is a clear description of how the method works practically and in theory, and (4) the relevance to regulatory applications is clear. If they receive that, the process of validation will be very much faster. Apart from their role, Dr. Gribaldo suggests that defining the method description, the protocol definition, and the characterization of test system will speed up both their processes and those of other organizations, and those include organizations involved with acceptance.

Another process that is important to take note of is the development of standards. From the perspective of EURL ECVAM, they reportedly receive many submissions they term as ''Me Too'' methods—methods that predict similar *in vivo* endpoints. As a result, EURL ECVAM developed a series of performance criteria to single out techniques that might have value.

IATA approaches—integrated approaches to testing and assessment—address the change that was seen following the introduction of the cosmetic regulations and the ban on animal testing. Namely, this involved a move away from single methods for single endpoints. Repeated-dose, chronic toxicity, oral discomfort, and toxicokinetic studies are all examples of studies that were needed following legislative changes. As a result of this, EURL ECVAM has tried to promote a more integrated approach toward validation—and that means combining data from multiple sources that include not only *in vitro* methods but also computational, epidemiological studies, existing animal data, and read-across methodologies.

In conclusion, Dr. Gribaldo said: ''It is important for us that when you propose a test or an approach, whether it is a single approach or multiple methods, that what you propose is clear scientifically and clear from the perspective of regulatory context(s).''

Expert panel discussion

Dr. Peter Kearns (OECD) and Dr. Gribaldo (EURL ECVAM) both touched on the surprising fact that while inhalation toxicology is a priority area for governments (air pollution, tobacco smoking being prime examples), little has been done in terms of validation of methods relating to inhalation toxicology. They particularly touched on the fact that animal use in this area still remains high, despite the fact that such animal-based experiments are widely questionable from many perspectives.

Dr. Gribaldo raised more questions with respect to the area of inhalation toxicology. In particular, why no method relating to inhalation toxicology has ever been submitted to them (EURL ECVAM) for validation remains unclear.

The expert panel discussion continued with Dr. Kearns of the OECD explaining their role in validation and method development and the regulatory context in which the process sits.

OECD test guidelines, while providing standardized approaches to assess the effects of chemicals on human health and the environment, are covered by the mutual acceptance of data system. This means that once a test guideline is accepted and used in a member state to generate data for regulatory purposes and under conditions that meet OECD Good Laboratory Practice guidelines, that data should then be accepted in all OECD countries. Legally binding instruments are in place to ensure this happens.

As Dr. Kearns explained, it is therefore very important for the OECD and the representatives of the regulatory authorities of the 34 member countries to ensure that methods used for safety testing are validated correctly. For example, if a party approaches a regulatory authority with data generated with an OECD test guideline, it is legally obliged to accept that data.

This is one of the major reasons why validation of methods can take a long time. All methods that have an OECD test guideline have been developed on a consensus basis by representatives of each individual member country and associated technical experts. Each individual method proposed for validation is usually presented by a member state (in Europe, the European Commission [EU] can also propose methods for validation) to the OECD with a validation package. There then follows a process in which each individual country has to be convinced that a validation is worthwhile. The main reason for this is that validation is time-consuming and can be costly. Questions around priorities, quality, and costs will likely all be considered.

In short, according to Dr. Kearns, governments have to have faith in the quality of methods before they can be used for regulatory purposes. Validation is therefore important to get right, but it is important to note that through the necessary consensus, building process and practical execution of studies can be a long and costly process.

Dr. Jean-Marc Aublant of the Laboratoire National de Metrologie et d'Essais (LNE), France, outlined the role of the ISO and the voluntary consensus-based standards they develop to support legislation, regulation, product development, and many other areas. Proposed ISO standards necessarily have to conform to a number of strict requirements and are usually developed over a period of time up to a maximum of 3 years. Proposed standards are usually submitted by national standard agencies. The process usually requires that data relating to round-robin validation studies be submitted in the first instance—proof that the method is scientifically validated in terms of reproducibility, repeatability, accuracy, precision, and so on. A metrology checklist is used to assess methods in the first instance along with questions about relevance, cost, and other such items. Once all these requirements are satisfied, the process of standard development can start. Dr. Aublant reiterated that because standards are used in different ways by different organizations, their methods of assessment are necessarily strict. This may at times seem unnecessary from an academic perspective. However, through experience, the ISO has found it necessary if standards are to prove useful for end users.

While Dr. Kearns and Dr. Aublant illustrated the perspectives of OECD and ISO on the validation process and in particular validation for regulatory purposes, a commercial perspective perhaps starts to illustrate a different issue that ALI *in vitro* modeling technology may face—the question of what to validate.

Dr. Samuel Constant is COO and co-founder of Epithelix Sarl, Switzerland, a company that specializes in the manufacture of 3D human tissue cell models that mimic various parts of the respiratory tract. While reportedly they have had some success developing and marketing their models, two key issues have emerged over the last 10 years. The first, according to Dr. Constant, is that accessing the regulatory process has proved challenging for a start. However, more importantly from his perspective, he sees an ongoing debate around what models to develop and even whether it is realistic to expect *in vitro* models to offer one single solution for inhalation toxicology assessments. In his opinion, one model is never likely to be enough since inhalation toxicology covers both local and systemic effects and in a sense should be compartmentalized to realistically assess endpoints. There are so many possible endpoints that can be measured (e.g., irritation, inflammation, carcinogenesis, fibrosis, and sensitization), suggesting it is very unlikely that there will be one model. According to Dr. Constant, there is a very real need to describe what we have available now, what can therefore be answered, and what is needed in the near future in terms of a toolbox to answer the most pressing research questions. In his opinion, at the moment, there is no toolbox available to validate in the first place.

Next Dr. Silvia Diabaté of the Karlsruhe Institute of Technology, Germany, described her academic group's experience with ALI *in vitro* inhalation models. According to Dr. Diabaté, they have been using such models now for several years and in particular to investigate the effects of nanomaterials on lung cells. In her opinion, it is important to have a good approach to aerosol generation and their characterization because aerosols based on different materials are so different. Particle size distribution, number, and mass concentration of the aerosol are all important for determining dose—something that in her opinion is not always routinely performed and thus not standard in the field. The different exposure systems all still need to be adequately investigated and compared with each other. For example, the deposition efficiency, exposure time, and dose rate can differ dramatically between various ALI systems, and hence, the biological response might be entirely altered. Particularly dose/response effects and underlying mechanisms of toxicity after exposure at the ALI are poorly understood. Going further she stated that in her opinion, it is important to test different cell systems and endpoints and that these should all be as close as possible to human physiology and if necessary human disease. In her opinion there are still many open questions about these systems and approaches, not least from the perspective of validation.

Ms. Antoinette de Groot from Solvay (a chemicals company) described her perspective as someone investigating whether to use ALI *in vitro* models. According to Ms. de Groot, their intention is to use such models for both research purposes and also possibly in regulatory assessments. However, in both cases there are issues due to a general lack of validation, which is making their decision to invest difficult. The sheer number of models available is also proving a challenge to navigate, with seemingly numerous organizations developing solutions that each seems to have advantages and disadvantages.

Echoing Ms. de Groot and Dr. Constant, Dr. Detlef Ritter of Frauhofer ITEM, Germany, tried to put some perspective onto the current diversity of methods available. The reasons he says there are so many and it comes down to the sheer numbers of variables, unknowns, endpoints, and biological effects that are being studied. It is therefore hard to imagine a scenario where even a small range of models validated to OECD guideline levels will be sufficient to assess all these different questions. It is therefore possible that such a strategy for this field is not in fact correct. Continuing, he suggested that standardized validation or performance criteria might be considered and that each individual method/laboratory uses these to describe the level of validation of their own methods.

The discussion continued with Dr. Kearns highlighting the work the OECD has done on nanomaterials and the development of test guidelines for that area. Accordingly, they started from the perspective of existing test guidelines for other chemicals and asked whether they were suitable for assessing nanomaterials or not. However, according to Dr. Kearns, an unexpected outcome was they were able to establish through that process what the priorities of their members (i.e., the governments of the 34 member states) actually were. And, according to Dr. Kearns, inhalation toxicology was more or less the top priority. Thus, in the process of assessing the test guidelines for nanomaterials, they found that many of the methods recommended were top priority, very expensive, and largely focused on animal-based approaches (which in itself raised questions around animal welfare). This then suggests there is a real need (demand) for *in vitro* methods in this area but that there are clear issues in getting them validated for regulatory purposes. It could be that there are too many options available or that the methods might be divided up according to endpoints to then enable prioritization. Nevertheless, Dr. Kearns was encouraged by the developments and suggested that the field should push for more meaningful validation studies for regulatory purposes.

Dr. Laura Gribaldo (EURL ECVAM) supported Dr. Kearns views by highlighting that the new EU legislation will require the need for validation of *in vitro* methods for inhalation toxicology. In her opinion, it is perhaps surprising that so little progress has been made in terms of validation of such *in vitro* methods, given the importance of the area from a health perspective. It may have been the case that methods simply did not exist previously and therefore validation did not feature in the mind-sets of either the research scientists developing the methods or regulatory authorities that might find a use for the methods for their tasks. However, that is not the case now, and as such, it seems reasonable to consider validation for the techniques, according to Dr. Gribaldo. To achieve this, she suggests the first steps likely require the identification of methods that could represent priority areas and that the validation and promotion of techniques should proceed. The most likely approach that could work would be to organize a network to define strategy, combine previously available information (and that might include non-*in vitro* information, such as *in silico* and *in vivo* evidence), and to define and execute trials, such as ring-trials, to start the validation process. This might involve a single test or a battery of tests, but the key, according to Dr. Gribaldo, is to get started on the process. Getting funding will obviously be important but will be key to having harmonized methods for the field of ALI *in vitro* inhalation

methods. It will also be important to define protocols, training needs, methods, and so on, but once this is done, it should be possible to submit the method for validation for regulatory purposes (presumably to EURL ECVAM).

In response to this, Dr. Ritter emphasized that finding one method (or more?) will be very difficult for the purposes of validation. According to him, for one material/chemical, the target might be the nose, lungs, or the alveolar, and that means there is never likely to be one singular endpoint—and that means a large range of methods will be needed. Rebutting Dr. Gribaldo, he said that it is probably too simplistic to expect a group to get together to plan out a study around one single method. Defining which method to validate, among the myriad of other methods available, is a core problem, according to Dr. Ritter.

Dr. Aublant (of LNE, representing ISO) followed up and emphasized the issues that were highlighted by Dr. Kearns (OECD)—namely, that priority setting should be a key target for the area. According to Dr. Aublant, whether or not a method is validated, a key issue is that correlations between what happens in humans, and in animals or cells, normally ends up in a gray zone of believable evidence. Sometimes the correlations are good between *in vitro* and *in vivo*. However, most of the time this is not the case. On that basis, Dr. Aublant reiterated that validation based on solid *in vivo*/*in vitro* correlations should be a prime consideration in any future study, particularly from the aspect of risk/benefit assessments. In particular, the issue, according to him, is that if there are little or no *in vivo* data, there is very little basis on which to establish an *in vitro* method.

In response to this, Dr. Ritter raised the point that whatever the target of analysis (i.e., the chemicals or compounds of interest), the reference points are weak. For inhalation toxicity studies, there are largely no reference points on which to base the potential methods and that are based on human studies. Where there are reference points, they tend to be based on animal models. This issue is key to the development of the area, according to Dr. Ritter.

Dr. Gribaldo supported this position, highlighting that for EURL ECVAM, they have had the same problem in that little human data have ever been available to root the alternative *in vitro* methods to human-derived data. Indeed, previous animal studies, that many methods are notionally based on for their effectiveness, are historic and likely not the most optimally designed. This, according to Dr. Gribaldo, is a constant challenge within the community involved in validation. Extrapolation of *in vitro* data to *in vivo* models is likely to be fraught with issues simply because the data derived from the initial experiments on animals and possibly humans (depending on the area) might well be compromised. However, in some areas, it may be possible to base the methods, in part, on epidemiological data. In this respect, occupational data may well be an option for some methods since such data may well be collected for legal reasons and also on a regular basis.

In subsequent discussions, it became clear that determining exactly what to validate is a core issue. As well as there being many different methods to assess endpoints, there are also many different types of apparatus and cells used in different steps of an experiment. For example, there are many different solutions available for generating aerosols, irrespective of what cell type is used. A question was therefore raised over whether these systems should also be individually validated. There was also a call for more collaboration between laboratories to standardize methods to allow more meaningful comparisons to be made. At the moment, this is reportedly hard to do since each laboratory uses slightly different methods for what are essentially the same experiments.

Dr. Aublant reiterated that standardized protocols are vital to allow meaningful comparisons to be made, giving an example of his experiences in relation to the physical characterization of nanomaterials. While he did agree that many different protocols might be needed for the assessment of the many endpoints in inhalation toxicology, there is still a need for such methods to be comparable. This might mean developing a reference method or some kind of standard that would allow laboratories to compare data.

A key point that emerged in the discussion is that there must be a balance struck between feasibility of validation and relevance of a method to an endpoint. Strikingly diverse opinions emerged on this, with a clear distinction between some being interested mostly in comparing endpoints and others more interested in ensuring methods are validated. Dr. Gribaldo highlighted, for example, that a method may be highly relevant for a particular endpoint, but if the cost of validation is too high for anyone to afford, it makes no sense to try to move forward. She highlighted that there will always be a fine balance between relevance to an endpoint, feasibility, and cost of validation and also the chances of wide adoption of a method once validated. This can also apply to building blocks of wider systems (presumably referring to different parts of ALI systems—exposure mechanisms, cells, analysis).

Dr. Kooter (TNO) went further suggesting that there are so many building blocks available, and that it is unlikely any one method will suffice for inhalation toxicology endpoints. Dr. Constant (Epithelix) later supported this view, suggesting that defining what needs validating is likely to be the first step for the field. Translocation and inflammation could be good first steps here, he said. However, he also reiterated that variability in cells should be expected. According to Dr. Kooter, data emerging from some collaborative research projects suggest considerable variability should be expected between different laboratories when cells are used in even simple experiments. Also, in more complicated methods, it is probably unwise to expect similar outcomes to those found in simple cell cultures.

Based on this, it might therefore be interesting to adopt an internal standard that is widely accepted by the community. This would go someway toward addressing the issue of comparability between experiments, if not validation. And, given the emerging doubt over whether validation will ever be achievable, this may go someway toward harmonizing approaches to achieve at least some comparability.

As with all models, limitations exist. And so, a question over whether ALI *in vitro* inhalation models are "better" than other approaches was likely to surface at such a workshop. At least from a conceptual standpoint, there was general agreement that such models offer a more realistic exposure scenario than can be achieved with submerged cells—most cells in the respiratory tract are exposed directly to air *in vivo*. ALI models have better sensitivity and selectivity

than single-cell cultures.[8] However, it is difficult to state that this is the case for all nanomaterials because such analyses have not been completed. Reproducibility and comparison of outcomes *in vivo* are yet to be completed. However, the capability to do such experiments exists—it was suggested on two occasions that worldwide, possibly 40 or even 100 laboratories do use ALI *in vitro* inhalation models of some description.

It is clear that guidance is needed on how to set up validation and what outcomes should be reported. Indeed, guidelines on aerosol generation, characterization of aerosols, exposure, cell systems, and analytics, and in particular their subsequent reporting, should be defined as a matter of urgency.

In terms of validation, it is important that laboratories use the same protocols and particularly define the dose, which together with exposure duration defines an endpoint. In this way, it should be possible for a set of laboratories to validate a method based on one or two substances.

A suggestion then followed that a starting point could also be based on whether good human *in vivo* data exist—for example, good data already exist on inflammatory responses in humans to titanium dioxide exposure. It would then ''simply'' be a question of testing whether a (series of) method(s) could detect the same effects and at what dose. This should mean it would be possible to define which system is the most sensitive—and whether it can detect effects at biologically relevant doses—and therefore defining which system should then proceed to full validation. As it is possible to quantify doses in ALI systems, this removes a major impediment that is suffered by submerged cultures, in that, dose in those systems is difficult to quantify in a meaningful way.

In a separate development, Dr. Aublant highlighted that a full standardization of characterization methods for the size of nanoparticles is underway in which a full set of protocols is now available. Twelve laboratories are now involved in the standardization program. Everything was standardized in terms of particles, methods, techniques, and so on. It is expected that this work will form the basis of an ISO standard and is expected to be published in the coming months. This may well be of interest to users of ALI *in vitro* inhalation models.

Dr. Hubert concluded by suggesting that while many methods exist now, it is likely that convergence of these will occur to the point where two to three methods may suffice. Whether that turns out to be the case though will depend on whether the field can come together and work on the issues surrounding validation together.

Supplementary References

1. Zuo YY, Veldhuizen RAW, Neumann AW, et al. Current perspectives in pulmonary surfactant—inhibition, enhancement and evaluation. Biochim Biophys Acta 2008:1778; 1947–1977.
2. Duch P, Nørgaard AW, Hansen JS, et al. Pulmonary toxicity following exposure to a tile coating product containing alkylsiloxanes. A clinical and toxicological evaluation. Clin Toxicol (Phila) 2014:52;498–505.
3. Sørli JB, Da Silva E, Bäckman P, et al. A proposed *in vitro* method to assess effects of inhaled particles on lung surfactant function. Am J Respir Cell Mol Biol 2016:54;306–311.
4. Larsen ST, Dallot C, Larsen SW, et al. Mechanism of action of lung damage caused by a nanofilm spray product. Toxicol Sci 2014:140;436–444.
5. Sørli JB, Huang Y, Da Silva E, et al. Prediction of acute inhalation toxicity using *in vitro* lung surfactant inhibition. ALTEX 2018:35;26–36.
6. Kim JS, Peters TM, O'Shaughnessy PT, et al. Validation of an *in vitro* exposure system for toxicity assessment of air-delivered nanomaterials. Toxicol In Vitro 2013:27; 164–173.
7. Xie Y, Williams NG, Tolic A, et al. Aerosolized ZnO nanoparticles induce toxicity in alveolar type II epithelial cells at the air-liquid interface. Toxicol Sci 2012:125;450–461.
8. Loret T, Peyret E, Dubreuil M, et al. Air-liquid interface exposure to aerosols of poorly soluble nanomaterials induces different biological activation levels compared to exposure to suspensions. Part Fibre Toxicol 2016:13;21.
9. Ceder R, Merne M, Staab CA, et al. The application of normal, SV40 T-antigen-immortalised and tumour-derived oral keratinocytes, under serum-free conditions, to the study of the probability of cancer progression as a result of environmental exposure to chemicals. Altern Lab Anim 2007:35; 621–639.
10. Hansson A, Bloor BK, Haig Y, et al. Expression of keratins in normal, immortalized and malignant oral epithelia in organotypic culture. Oral Oncol 2001:37;419–430.
11. Hansson A, Bloor BK, Sarang Z, et al. Analysis of proliferation, apoptosis and keratin expression in cultured normal and immortalized human buccal keratinocytes. Eur J Oral Sci 2003:111;34–41.
12. Kimball JR, Nittayananta W, Klausner M, et al. Antimicrobial barrier of an *in vitro* oral epithelial model. Arch Oral Biol 2006:51;775–783.
13. Klausner M, Ayehunie S, Breyfogle BA, et al. Organotypic human oral tissue models for toxicological studies. Toxicol In Vitro 2007:21;938–949.
14. Koschier F, Kostrubsky V, Toole C, et al. *In vitro* effects of ethanol and mouthrinse on permeability in an oral buccal mucosal tissue construct. Food Chem Toxicol 2011:49; 2524–2529.
15. Moharamzadeh K, Colley H, Murdoch C, et al. Tissue-engineered oral mucosa. J Dent Res 2012:91;642–650.
16. Schlage WK, Iskandar AR, Kostadinova R, et al. *In vitro* systems toxicology approach to investigate the effects of repeated cigarette smoke exposure on human buccal and gingival organotypic epithelial tissue cultures. Toxicol Mech Methods 2014:24;470–487.
17. Vande Vannet B, Hanssens J-L, Wehrbein H. The use of three-dimensional oral mucosa cell cultures to assess the toxicity of soldered and welded wires. Eur J Orthod 2007: 29;60–66.
18. Walle T, Walle UK, Sedmera D, et al. Benzo[A]pyrene-induced oral carcinogenesis and chemoprevention: studies in bioengineered human tissue. Drug Metab Dispos 2006: 34;346–350.
19. Balharry D, Sexton K, BéruBé KA. An *in vitro* approach to assess the toxicity of inhaled tobacco smoke components: nicotine, cadmium, formaldehyde and urethane. Toxicology 2008:244;66–76.
20. Mathis C, Poussin C, Weisensee D, et al. Human bronchial epithelial cells exposed *in vitro* to cigarette smoke at the air-liquid interface resemble bronchial epithelium from human smokers. Am J Physiol Lung Cell Mol Physiol 2013:304; L489–L503.
21. Maunders H, Patwardhan S, Phillips J, et al. Human bronchial epithelial cell transcriptome: gene expression changes following acute exposure to whole cigarette smoke *in vitro*.

Am J Physiol Lung Cell Mol Physiol 2007:292;L1248–L1256.
22. Gualerzi A, Sciarabba M, Tartaglia G, et al. Acute effects of cigarette smoke on three-dimensional cultures of normal human oral mucosa. Inhal Toxicol 2012:24;382–389.
23. Zanetti F, Sewer A, Mathis C, et al. Systems toxicology assessment of the biological impact of a candidate modified risk tobacco product on human organotypic oral epithelial cultures. Chem Res Toxicol 2016:29;1252–1269.
24. Zanetti F, Titz B, Sewer A, et al. Comparative systems toxicology analysis of cigarette smoke and aerosol from a candidate modified risk tobacco product in organotypic human gingival epithelial cultures: a 3-day repeated exposure study. Food Chem Toxicol 2017:101;15–35.
25. BéruBé K, Aufderheide M, Breheny D, et al. *In vitro* models of inhalation toxicity and disease. The report of a FRAME workshop. Altern Lab Anim 2009:37;89–141.
26. Chi AC, Appleton K, Henriod JB, et al. Differential induction of CYP1A1 and CYP1B1 by benzo a pyrene in oral squamous cell carcinoma cell lines and by tobacco smoking in oral mucosa. Oral Oncol 2009:45;980–985.
27. Spivack SD, Hurteau GJ, Jain R, et al. Gene-environment interaction signatures by quantitative mRNA profiling in exfoliated buccal mucosal cells. Cancer Res 2004:64;6805–6813.
28. Vondracek M, Xi Z, Larsson P, et al. Cytochrome P450 expression and related metabolism in human buccal mucosa. Carcinogenesis 2001:22;481–488.
29. Knight DA, Holgate ST. The airway epithelium: structural and functional properties in health and disease. Respirology 2003:8;432–446.
30. Huang S, Wiszniewski L, Constant S. The use of *in vitro* 3D cell models in drug development for respiratory diseases. In: *Drug Discovery and Development-Present and Future*. IM Kapetanovic (ed); InTech; 2011.
31. Huang S, Boda B, Vernaz J, et al. Establishment and characterization of an *in vitro* human small airway model (SmallAir). Eur J Pharm Biopharm 2017:118;68–72.
32. Constant S, Huang S, Wisniewski L, et al. Advanced human *in vitro* models for the discovery and development of lung cancer therapies. In: *Drug Discovery and Development-From Molecules to Medicine*. O Vallisuta, S Olimat (eds); InTech; 2015.
33. Mas C, Boda B, CaulFuty M, et al. Antitumour efficacy of the selumetinib and trametinib MEK inhibitors in a combined human airway–tumour–stroma lung cancer model. J Biotechnol 2015:205;111–119.
34. Mas C, Boda B, Caul Futy M, et al. Establishment of a tumour-stroma airway model (OncoCilAir) to accelerate the development of human therapies against lung cancer. Altern Lab Anim 2016:44;479–485.
35. Maynard AD, Aitken RJ, Butz T, et al. Safe handling of nanotechnology. Nature 2006:444;267–269.
36. Oberdörster G, Maynard A, Donaldson K, et al. Principles for characterizing the potential human health effects from exposure to nanomaterials: elements of a screening strategy. Part Fibre Toxicol 2005:2;8.
37. Oberdörster G, Stone V, Donaldson K. Toxicology of nanoparticles: a historical perspective. Nanotoxicology 2007:1;2–25.
38. Hartung T. From alternative methods to a new toxicology. Eur J Pharm Biopharm 2011:77;338–349.
39. Rothen-Rutishauser BM, Kiama SG, Gehr P. A three-dimensional cellular model of the human respiratory tract to study the interaction with particles. Am J Respir Cell Mol Biol 2005:32;281–289.
40. Lehmann AD, Daum N, Bur M, et al. An *in vitro* triple cell co-culture model with primary cells mimicking the human alveolar epithelial barrier. Eur J Pharm Biopharm 2011:77; 398–406.
41. Lenz AG, Karg E, Lentner B, et al. A dose-controlled system for air-liquid interface cell exposure and application to zinc oxide nanoparticles. Part Fibre Toxicol 2009:6;32.
42. Bachler G, Losert S, Umehara Y, et al. Translocation of gold nanoparticles across the lung epithelial tissue barrier: combining *in vitro* and in silico methods to substitute in vivo experiments. Part Fibre Toxicol 2015:12;18.
43. Brandenberger C, Rothen-Rutishauser B, Mühlfeld C, et al. Effects and uptake of gold nanoparticles deposited at the air-liquid interface of a human epithelial airway model. Toxicol Appl Pharmacol 2010:242;56–65.
44. Herzog F, Clift MJD, Piccapietra F, et al. Exposure of silver-nanoparticles and silver-ions to lung cells *in vitro* at the air-liquid interface. Part Fibre Toxicol 2013:10;11.
45. Lenz AG, Karg E, Brendel E, et al. Inflammatory and oxidative stress responses of an alveolar epithelial cell line to airborne zinc oxide nanoparticles at the air-liquid interface: a comparison with conventional, submerged cell-culture conditions. Biomed Res Int 2013:12;1–12.
46. Stoehr LC, Endes C, Radauer-Preiml I, et al. Assessment of a panel of interleukin-8 reporter lung epithelial cell lines to monitor the pro-inflammatory response following zinc oxide nanoparticle exposure under different cell culture conditions. Part Fibre Toxicol 2015:12;29.
47. Endes C, Mueller S, Kinnear C, et al. Fate of cellulose nanocrystal aerosols deposited on the lung cell surface *in vitro*. Biomacromolecules 2015:16;1267–1275.
48. Endes C, Schmid O, Kinnear C, et al. An *in vitro* testing strategy towards mimicking the inhalation of high aspect ratio nanoparticles. Part Fibre Toxicol 2014:11;40.
49. Chortarea S, Clift MJD, Vanhecke D, et al. Repeated exposure to carbon nanotube-based aerosols does not affect the functional properties of a 3D human epithelial airway model. Nanotoxicology 2015:9;983–993.
50. Gangwal S, Brown JS, Wang A, et al. Informing selection of nanomaterial concentrations for ToxCast *in vitro* testing based on occupational exposure potential. Environ Health Perspect 2011:119;1539–1546.
51. Hiemstra PS, McCray PB, Jr., Bals R. The innate immune function of airway epithelial cells in inflammatory lung disease. Eur Respir J 2015:45;1150–1162.
52. Mertens TCJ, Hiemstra PS, Taube C. Azithromycin differentially affects the IL-13-induced expression profile in human bronchial epithelial cells. Pulm Pharmacol Ther 2016: 39;14–20.
53. Zuyderduyn S, Ninaber DK, Schrumpf JA, et al. IL-4 and IL-13 exposure during mucociliary differentiation of bronchial epithelial cells increases antimicrobial activity and expression of antimicrobial peptides. Respir Res 2011:12;59.
54. Amatngalim GD, Schrumpf JA, Henic A, et al. Antibacterial defense of human airway epithelial cells from chronic obstructive pulmonary disease patients induced by acute exposure to nontypeable *Haemophilus influenzae*: modulation by cigarette smoke. J Innate Immun 2017:9;359–374.
55. Amatngalim GD, van Wijck Y, de Mooij-Eijk Y, et al. Basal cells contribute to innate immunity of the airway epithelium through production of the antimicrobial protein RNase 7. J Immunol 2015:194;3340–3350.

56. Zarcone MC, Duistermaat E, van Schadewijk A, et al. Cellular response of mucociliary differentiated primary bronchial epithelial cells to diesel exhaust. Am J Physiol Lung Cell Mol Physiol 2016:311;L111–L123.
57. Hiemstra PS, Grootaers G, van der Does AM, et al. Human lung epithelial cell cultures for analysis of inhaled toxicants: lessons learned and future directions. Toxicol In Vitro 2017:47;137–146.
58. Zarcone MC, van Schadewijk A, Duistermaat E, et al. Diesel exhaust alters the response of cultured primary bronchial epithelial cells from patients with chronic obstructive pulmonary disease (COPD) to non-typeable *Haemophilus influenzae*. Respir Res 2017:18;27.
59. van der Burg B, Winter R, Man H-Y, et al. Optimization and prevalidation of the *in vitro* AR CALUX method to test androgenic and antiandrogenic activity of compounds. Reprod Toxicol 2010:30;18–24.
60. European Commission. REACH—chemicals—environment—European Commission. 2016. Available at: http://ec.europa.eu/environment/chemicals/reach/reach_en.htm (last accessed March 12, 2018).
61. European Commission. Cosmetics—legislation—European Commission. 2016. Available at: http://ec.europa.eu/growth/sectors/cosmetics/legislation_en (last accessed March 12, 2018).
62. European Medicines Agency. International-standard organisations. 2016. Available at: http://www.ema.europa.eu/ema/index.jsp?curl=pages/partners_and_networks/general/general_content_000227.jsp&mid=WC0b01ac05801df740 (last accessed March 12, 2018).
63. European Directorate for the Quality of Medicines/Council of Europe. European Pharmacopoeia (Ph. Eur. 9th ed.). 2017. Available at: https://www.edqm.eu/en/european-pharmacopoeia-ph-eur-9th-edition (last accessed March 12, 2018).
64. OECD. OECD Guidelines for the testing of chemical. 2016. Available at: http://www.oecd.org/chemicalsafety/testing/oecdguidelinesforthetestingofchemicals.htm (last accessed March 12, 2018).
